# Transplanting rejuvenated blood stem cells extends lifespan of aged immunocompromised mice

**DOI:** 10.1038/s41536-022-00275-y

**Published:** 2022-12-29

**Authors:** Sara Montserrat-Vazquez, Noelle J. Ali, Francesca Matteini, Javier Lozano, Tu Zhaowei, Eva Mejia-Ramirez, Gina Marka, Angelika Vollmer, Karin Soller, Mehmet Sacma, Vadim Sakk, Loris Mularoni, Jan Philipp Mallm, Mireya Plass, Yi Zheng, Hartmut Geiger, M. Carolina Florian

**Affiliations:** 1 Stem Cell Aging Group, Regenerative Medicine Program, The Bellvitge Institute for Biomedical Research (IDIBELL), L’Hospitalet de Llobregat, Barcelona, Spain; 2 Program for advancing the Clinical Translation of Regenerative Medicine of Catalonia, P-CMR[C], L’Hospitalet de Llobregat, Barcelona, Spain; 3 Institute of Molecular Medicine, University of Ulm, Ulm, Germany; 4 Division of Experimental Hematology and Cancer Biology, Cincinnati Children’s Hospital Medical Center, Cincinnati, OH USA; 5 Center for Networked Biomedical Research on Bioengineering, Biomaterials and Nanomedicine (CIBER-BBN), Madrid, Spain; 6 DKFZ, Heidelberg, Germany; 7 Gene Regulation of Cell Identity Group, Regenerative Medicine Program, The Bellvitge Institute for Biomedical Research (IDIBELL), L’Hospitalet de Llobregat, Barcelona, Spain

**Keywords:** Ageing, Regeneration, Haematopoietic stem cells, Muscle stem cells

## Abstract

One goal of regenerative medicine is to rejuvenate tissues and extend lifespan by restoring the function of endogenous aged stem cells. However, evidence that somatic stem cells can be targeted in vivo to extend lifespan is still lacking. Here, we demonstrate that after a short systemic treatment with a specific inhibitor of the small RhoGTPase Cdc42 (CASIN), transplanting aged hematopoietic stem cells (HSCs) from treated mice is sufficient to extend the healthspan and lifespan of aged immunocompromised mice without additional treatment. In detail, we show that systemic CASIN treatment improves strength and endurance of aged mice by increasing the myogenic regenerative potential of aged skeletal muscle stem cells. Further, we show that CASIN modifies niche localization and H4K16ac polarity of HSCs in vivo. Single-cell profiling reveals changes in HSC transcriptome, which underlie enhanced lymphoid and regenerative capacity in serial transplantation assays. Overall, we provide proof-of-concept evidence that a short systemic treatment to decrease Cdc42 activity improves the regenerative capacity of different endogenous aged stem cells in vivo, and that rejuvenated HSCs exert a broad systemic effect sufficient to extend murine health- and lifespan.

## Introduction

Aging is associated with loss of stem cell regenerative capacity in many tissues. A central goal of regenerative medicine is to restore or rejuvenate tissues by preserving the activity of endogenous stem cells over time. While it is generally accepted that stem cell exhaustion contributes to the decline of tissue homeostasis during aging and several promising strategies have been proposed targeting stem cells for tissue rejuvenation^1,2^, systemic rejuvenation remains a challenge. Moreover, it is still not completely clear to what degree do stem cells contribute to overall organism health- and lifespan^3^.

Cell polarity is critical for asymmetric cell division, which underlies the self-renewal and differentiation capacity of somatic stem cells over time^4,5^. Cdc42 is a small RhoGTPase that cycles between an active (GTP-bound) and an inactive (GDP-bound) state and plays a central role in cell polarity establishment in most organisms ranging from yeast to mammals^6,7^. With physiological aging, Cdc42-GTP levels increase in several tissues in mice and humans. The constitutive gain of function of Cdc42 in mice is achieved by genetic deletion of *Cdc42GAP* (or *Arhgap1* also known as *p50RhoGAP*), which is an ubiquitously expressed negative regulator of Cdc42, that catalyzes GTP hydrolysis by Cdc42 leading to Cdc42 inactivation. In *Cdc42GAP* knock-out mice, Cdc42-GTP is not hydrolyzed and levels of active Cdc42 persist elevated in the cells, without affecting activity levels of other small RhoGTPases. Importantly, *Cdc42GAP* knock-out results in a premature aging-like phenotype in mice that affects several organs and overall mouse fitness^8^. In detail, Cdc42GAP mice present with 2- to 3-fold higher Cdc42-GTP levels in different tissues and compared to wild-type littermates their lifespan is significantly shorter. They also show a reduction in body mass, loss of subdermal adipose tissue, severe lordokyphosis, muscle atrophy, osteoporosis, impaired wound-healing and hair regeneration, anemia, and other hematopoietic phenotypes. Consistently, the increased activity of Cdc42 with aging has also been shown to impair the function of several somatic stem cells in different tissues, and as a consequence, to negatively affect at least blood, skin, and intestine tissue homeostasis and regeneration^9–16^.

Recently, we reported that the systemic treatment of aged mice with a Cdc42 activity-specific inhibitor (CASIN)^17^ for only 4 consecutive days significantly extends their average and maximum lifespan^18^. Considering the importance of Cdc42 activity in cell polarity and for stem cell function^9–16^, here we investigated whether this brief systemic treatment with CASIN improves the capacity of endogenous aged stem cells to regenerate tissues and the extent to which this affects murine health- and lifespan.

## Results

### Systemic inhibition of Cdc42 activity improves aged mouse fitness

**Fig. 1:**
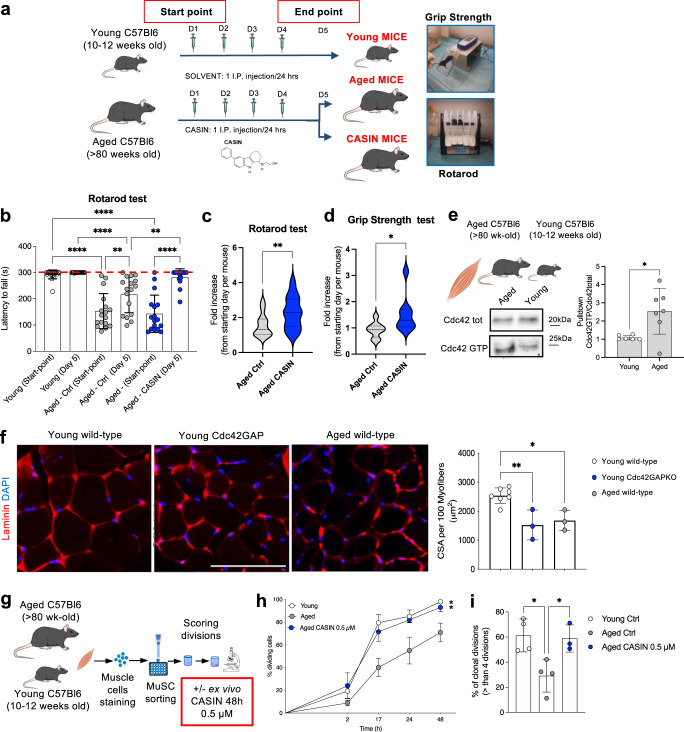
Systemic inhibition of Cdc42 activity improves aged mouse fitness. **a** Experimental design for the fitness training combined with the CASIN-treatment scheme. **b**–**d** Rotarod latency to fall, endurance measurement and grip strength test fold increase (Young, *n* = 20; Aged/CASIN, *n* = 17/15). The red dotted line in panel **b** represents the end of the experimental recording time (300 s). **e** Pulldown Cdc42GTP/Cdc42 total in muscle tissues (Young, *n* = 6; Aged, *n* = 7). All blots derive from the same experiment and were processed in parallel. **f** Representative histological analysis of skeletal muscle sections and CSA quantification. Scale bar, 300 µm. **g** Experimental scheme for single MuSC growth kinetics. **h** Growth curve of single MuSCs from young, aged and CASIN mice (*n* = 3). **i** Quantification of single MuSC clonal divisions on day 6 (Young, *n* = 4; Aged, *n* = 4; CASIN, *n* = 3). Columns are mean ± SD. *P < 0.05, **P < 0.01, ***P < 0.001, and ****P < 0.0001; one-way ANOVA, multiple comparison with Tukey test for **b**, **c**; *t*-test for **d**–**f**; Dunnett’s *vs* aged for **h**, **i**. Source data are provided as a Source Data file. Mouse cartoon: CreativeBucket:stock.adobe.com. Muscle cartoon: smart.servier.com.

To approach the question of how targeting Cdc42 activity eventually extends murine health- and lifespan, we started by considering that the constitutive gain of function of Cdc42 in mice (Cdc42GAP mice) results in a premature aging-like phenotype that affects overall mouse fitness^8^. Therefore, we wondered if, together with increased lifespan^18^, overall fitness of aged mice was improved by inhibiting Cdc42 activity. Systemic CASIN treatment was performed on >80-week-old C57Bl6 mice for 4 consecutive days (1 i.p. injection every 24 h) according to the previously published protocol^18^. As expected^19,20^, while overall locomotor activity was reduced in aged mice compared to the young ones, locomotor activity was markedly improved in the group of aged mice treated with CASIN (Supplementary Movie 1). To quantitatively estimate overall mouse fitness, we used the endurance assay and the grip strength test comparing young, aged, and aged CASIN-treated mice (indicated as ‘CASIN’ mice from now on; Fig. 1a). Results showed that CASIN mice remained significantly longer on the running wheel compared to aged control mice (Fig. 1b and Supplementary Movie 2) and they performed very similar to young mice. While we detected no changes in body weight (Supplementary Fig. 1a), CASIN treatment overall improved >2-fold mouse endurance and 1.5-fold grip strength compared to aged control mice (Fig. 1c, d). Together, we concluded that after the treatment with CASIN, aged mice improved their locomotor activity, endurance, and grip strength in line with an overall improvement of their healthspan.

### CASIN increases the regenerative potential of aged skeletal muscle stem cells

Next, we explored potential mechanisms which might mediate the fitness improvement of aged mice after inhibiting Cdc42 activity. Since endurance and grip strength are associated among others to improved skeletal muscle function^21,22^, we wondered if Cdc42 activity increases in this tissue with aging and if increased Cdc42 activity levels are sufficient to drive aging in the skeletal muscle. By pulldown and western blot assays, we detected a 2.1-fold increase in Cdc42-GTP levels in skeletal muscle cells harvested from aged mice (Fig. 1e and Supplementary Figure 2a). To gain further mechanistic support, we measured the myofiber cross-sectional area (CSA), which is correlated to myofiber number and mass and decreases in conditions of sarcopenia and aging, in aged, young wild-type and young Cdc42GAP mice (which have constitutive high level of active Cdc42). Consistently with the premature aging phenotype^8^, data showed significantly reduced myofiber cross-sectional area (CSA) in both Cdc42GAP and aged mice (Fig. 1f), suggesting an effect of increased Cdc42 activity in inducing premature aging of the skeletal muscle.

Skeletal muscle homeostasis tightly depends on skeletal muscle stem cells (MuSCs), which are indispensable to preserve tissue regeneration over time^23,24^. With aging, a progressive loss in total MuSC pool size and a decline in both MuSC and skeletal muscle function and mass is observed^21,22,25,26^. Therefore, we wondered if increased Cdc42 activity is associated to a decline in MuSC function. While young Cdc42GAP mice showed no decline in MuSC number (Supplementary Figure 1b), constitutive activation of Cdc42 reduced the frequency of young MuSC divisions and decreased their myogenic colony capacity after 6 days of culturing (Supplementary Figure 1c–e). This data implies a role for increased Cdc42 activation in the impairment of MuSC function. Since Cdc42 activity is increased in aged skeletal muscle cells, we tested whether decreasing Cdc42-GTP levels in aged MuSCs affects their divisional kinetics and clonogenic capacity. Consequently, we treated FACS-sorted aged MuSCs in vitro with CASIN to specifically target and reduce Cdc42-GTP levels. We tested in vitro doses within the window of concentrations detected in serum according to the previously published in vivo treatment^18^ (dose range 0.5–5 µM) and assayed MuSC divisional kinetics and their myogenic colony capacity after 6 days of culturing. Consistently with our hypothesis, decreasing Cdc42 activity in aged MuSCs by ex vivo CASIN treatment already at 0.5 µM doses significantly increased divisional kinetics and myogenic colony capacity of aged stem cells, to resemble young MuSCs (Fig. 1g–i). Additionally, we performed myofiber isolation to count proliferating cell clusters^27,28^. We measured a reduced number of clusters with >10 cells on aged myofibers compared to young, which was partially restored when cultured in the presence of CASIN (Supplementary Fig. 1f, g). Therefore, we concluded that Cdc42 activity is increased in aged skeletal muscles, that increased Cdc42 activity triggers aging-like phenotypes in young skeletal muscle and MuSCs, and that ex viv*o* treatment of aged MuSCs with CASIN improved MuSC activity in vitro.

**Fig. 2:**
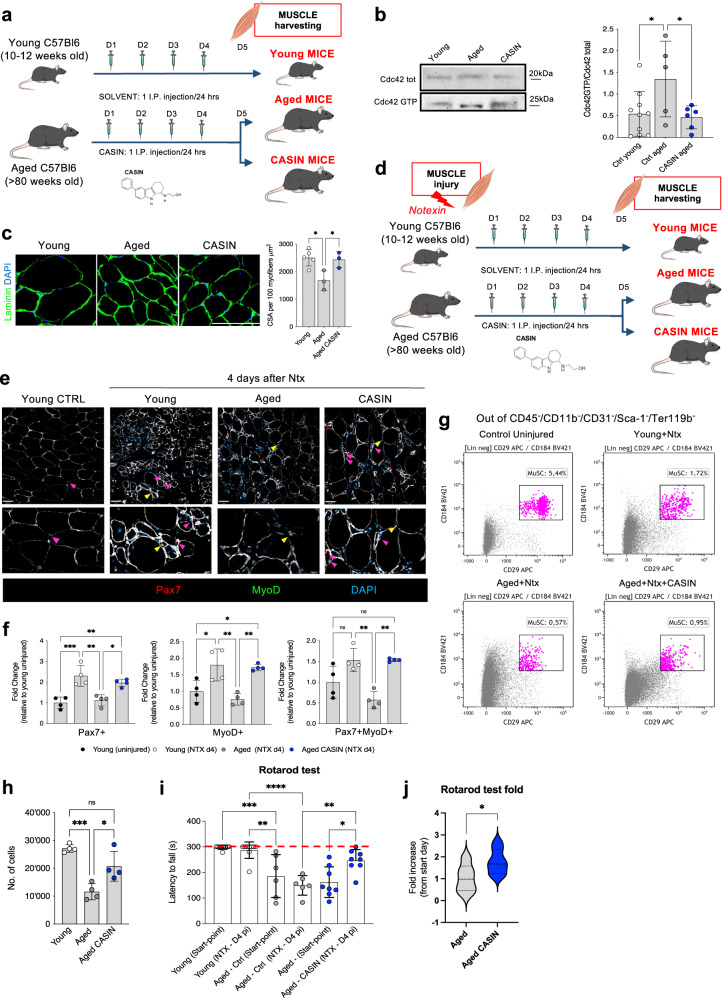
Systemic inhibition of Cdc42 activity improves the regenerative potential of aged MuSCs. **a** Experimental design. **b** Pulldown Cdc42GTP/Cdc42 (Young, *n* = 9; Aged, *n* = 5; CASIN, *n* = 6). All blots derive from the same experiment and were processed in parallel. **c** Immunofluorescent images and CSA quantification of myofibers in TA muscle. Scale bars, 200 µm. (Young, *n* = 5; Aged, *n* = 3; CASIN, *n* = 3). **d** Experimental scheme for Notexin+CASIN treatment. **e**, **f** Representative histology analyses and quantification of muscle cells positive for Pax7, MyoD, or both markers in the TA of Notexin±CASIN treated mice on day 4. Magenta arrowheads indicate Pax7^+^ MuSCs and yellow arrowheads indicate Pax7^+^MyoD^+^ MuSCs (*n* = 4). Scale bars, 42 µm. **g**, **h** Representative flow cytometry analysis and quantification of total MuSCs in Notexin±CASIN treated mice on day 4. (*n* = 4). **i**, **j** Rotarod latency to fall and fold change (Young, *n* = 9; Aged, *n* = 6; CASIN, *n* = 8). The red dotted line in panel **i** represents the end of the experimental recording time (300 s). Columns are mean ±SD. *P < 0.05, **P < 0.01, ***P < 0.001 and ****P < 0.0001; one-way ANOVA, multiple comparisons with Tukey test for **b**, **c**, **e**–**g**; *t*-test for **h**. Source data are provided as a Source Data file. Mouse cartoon: CreativeBucket:stock.adobe.com. Muscle cartoon: smart.servier.com.

Encouraged by this data, we hypothesized that the fitness improvement of aged mice after systemic CASIN treatment (Fig. 1b–d) could be, at least in part, associated to a reduction of Cdc42 activity in the skeletal muscle and in MuSCs in vivo. Thus, we repeated the systemic treatment of aged mice with CASIN and 24 h after the last injection we harvested the skeletal muscle tissue from their hindlimb muscles (*tibialis anterioris* (TA) and *gastrocnemius*) (Fig. 2a). Western blot and pull-down analyses confirmed a significant reduction of Cdc42 activity in the skeletal muscle tissue of CASIN mice to the level measured in young mice (Fig. 2b and Supplementary Fig. 2b). Histological myofiber CSA quantification displayed a significant increase in mean myofiber CSA area in CASIN compared to aged mice, which showed CSAs comparable to young mice (Fig. 2c). Further, to challenge the resident MuSCs and assess their regenerative capacity, we injected Notexin (Ntx) in the TA of young and aged mice and treated some of the aged mice with CASIN during the days immediately following the injury (Fig. 2d). Ntx is known to locally damage the myofibers and activate endogenous MuSCs to regenerate the tissue. Compared with the aged control group, CASIN mice showed a significant increase in the numbers of overall (Pax7^+^cells) and activated (Pax7^+^MyoD^+^ cells) MuSCs and of differentiating myoblasts (MyoD^+^ cells) at the site of injury (Fig. 2e, f). Consistently, we detected more MuSCs by flow cytometry analysis in the TA of Ntx+CASIN mice compared to aged Ntx control mice (Fig. 2g, h). Importantly, this increase in MuSC number and activation was mirrored by a functional health improvement since Ntx+CASIN mice remained significantly longer on the running wheel and significantly increased their endurance on the rotarod compared to before the treatment, while no changes were observed in aged Ntx control mice (Supplementary Fig. 3 and Fig. 2i, j). Therefore, systemic treatment with CASIN reduces Cdc42 activity and increases the regenerative potential of endogenous aged MuSC after injury, contributing to improving healthspan of aged mice.

### Systemic inhibition of Cdc42 activity increases hematopoietic stem cell polarity and restores stem cell localization in bone marrow of aged mice

**Fig. 3:**
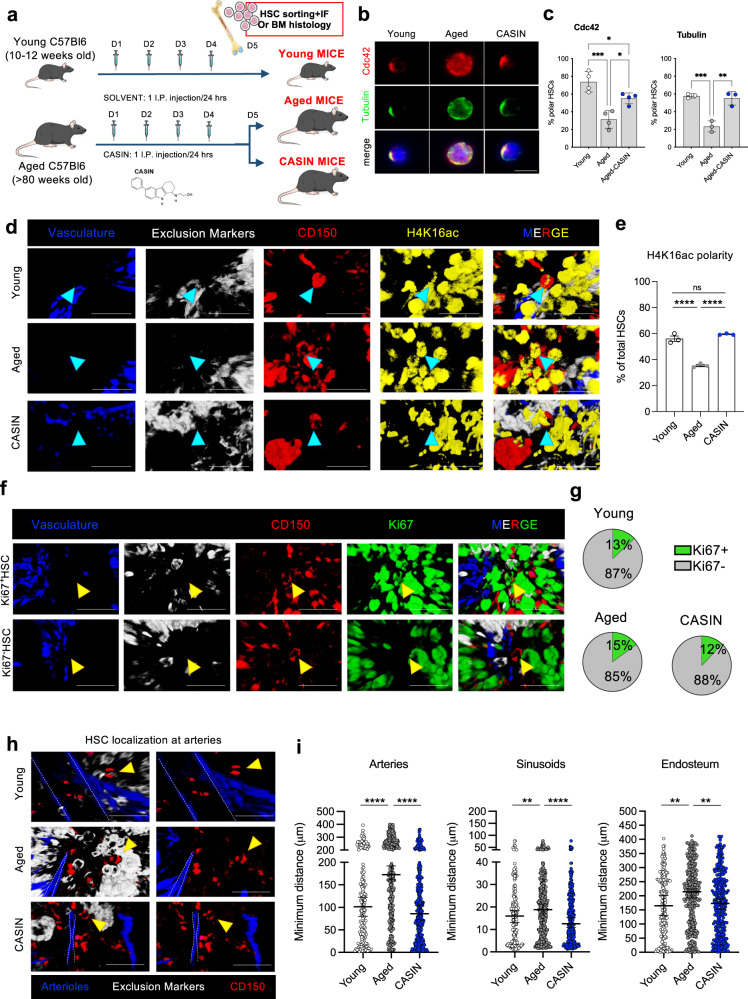
Systemic inhibition of Cdc42 activity increases hematopoietic stem cell (HSC) polarity and restores stem cell localization in BM of aged mice. **a** Experimental design. **b** Representative IF images of single FACS-sorted HSCs from young, aged, and CASIN mice stained for Cdc42 (red), tubulin (green) and the nucleus (DAPI, blue). Scale bars, 10 µm. **c** IF quantification of the percentages of HSCs polar for Cdc42 and tubulin (*n* = 4). Columns are mean ±SD. **d** Histological analysis of H4K16ac polarity in HSCs (arrowed). Scale bars, 20 µm. **e** Quantification of the percentage of HSCs polar for H4K16ac in young, aged, and CASIN mice (*n* = 3). Columns are mean ±SE. **f** Histological analysis of HSC proliferation rate by Ki67 staining. Scale bars, 20 µm. **g** Quantification of the percentage of HSCs Ki67^−^ and Ki67^+^ (*n* = 3). 203, 374, and 371 total HSCs for young, aged, and CASIN respectively were analyzed. **h** Quiescent HSC proximity to arteries in young, aged and CASIN mice. Scale bars, 35 µm. **i** Quantification of the minimum distance of HSCs to the closest artery, sinusoid, and endosteum (*n* = 3). Values are median with 95% CI. Columns are mean ±SE. *P < 0.05, **P < 0.01, ***P < 0.001 and ****P < 0.0001; one-way ANOVA, multiple comparisons with Tukey test for **c**, **e**; Mann–Whitney test two tails for **i**. Source data are provided as a Source Data file. Mouse cartoon: CreativeBucket:stock.adobe.com. Bone and cells cartoon: smart.servier.com.

Increased Cdc42 activity is associated to aging and rejuvenation of the hematopoietic system, which tightly depends on the activity of hematopoietic stem cells (HSCs)^9,10,12^. HSCs or blood stem cells reside in the bone marrow (BM) and preserve homeostasis of the hematopoietic system over time. Therefore, we wondered whether systemic CASIN treatment of aged mice affects directly blood stem cells. To address this, the BM of aged CASIN and control mice was harvested 24 h after the end of the treatment and hematopoietic stem and progenitor cells in BM were analyzed by different approaches. Since the important role of Cdc42 activity for cell polarity establishment in general^7^ and previous report showing loss of cell polarity in aged HSCs^9,10,14,29–33^, blood stem cells were sorted for cell polarity immunofluorescence (IF) staining (Fig. 3a). Some of the bones were also processed for whole-mount histological analyses to image HSCs directly within their BM microenvironment^34^. By IF, both Cdc42 and tubulin cell polarity were significantly increased in HSCs from CASIN mice (Fig. 3b, c). Whole-mount histological reconstruction of HSCs in high-resolution 3D fluorescence staining^34^ showed increased frequency of H4K16ac epigenetic polarity in stem cells of CASIN mice (Fig. 3d, e and Supplementary Fig. 4a, b). To note, H4K16ac levels and polarity have been previously associated with HSC regenerative capacity and chromatin architecture^29,35,36^. The data clearly supports that a systemic treatment with CASIN affects this epigenetic mark in HSCs (Fig. 3d, e). We detected no changes in HSC and hematopoietic progenitor cell frequency in BM of CASIN mice compared to aged control (Supplementary Figure 4c, d) by flow cytometry and based on histological Ki67 staining, we detected no changes in HSC proliferation rate across samples (Fig. 3f, g and Supplementary Fig. 4e). We and others showed previously that HSC localization in the BM is not random and is predictive of HSC function. While young quiescent HSCs lie close to BM arteriolar vessels and to the endosteum, in aged mice quiescent BM stem cells localize mainly close to sinusoidal vessels^37,38^. Interestingly, quiescent Ki67^−^ HSCs were found significantly closer to arteries and to the endosteum in young and CASIN mice than in aged mice (Fig. 3h, i and Supplementary Fig. 4f, g). Altogether, these data demonstrate that systemic CASIN treatment restores Cdc42 and H4K16ac polarity and the localization of quiescent aged HSCs to the levels seen in young mice.

### Systemic inhibition of Cdc42 activity increases the regenerative potential of aged blood stem cells

**Fig. 4:**
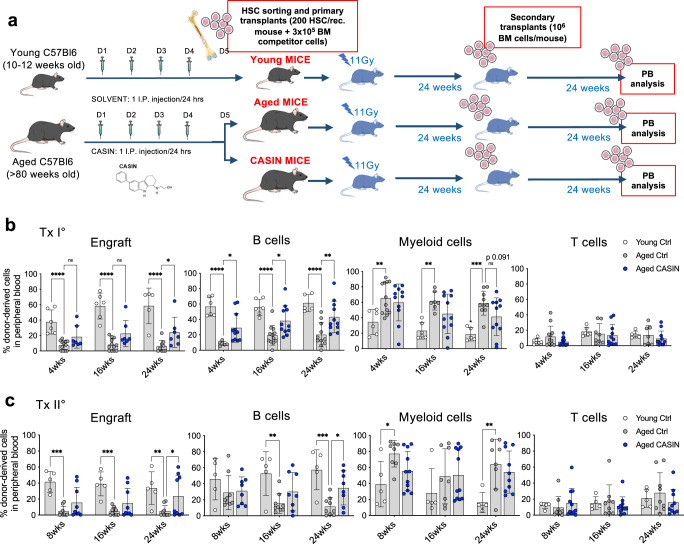
Systemic inhibition of Cdc42 activity increases the regenerative potential of aged blood stem cells. **a** Experimental design of serial competitive transplants in irradiated recipients. 200 sorted HSCs were transplanted alongside 3 × 10^5^ bone marrow competitor cells. PB: peripheral blood. **b**, **c** Flow cytometry quantification of peripheral blood profile of recipient mice in primary (Tx I°) and secondary (Tx II°) transplants. (Young, *n* = 5/6; Aged, *n* = 9/12; CASIN, *n* = 7/12). Columns are mean ±SD. *P < 0.05, **P < 0.01, ***P < 0.001 and ****P < 0.0001; one-way ANOVA, multiple comparisons with Dunnett’s test. Source data are provided as a Source Data file. Mouse cartoon: CreativeBucket:stock.adobe.com. Bone and cells cartoon: smart.servier.com.

Since aged HSCs in CASIN mice display an improved and youthful phenotype as for Cdc42 and H4k16ac polarity and BM localization, we decided to challenge their regenerative capacity by performing serial competitive transplantations into lethally irradiated recipient mice (Fig. 4a). Competitive transplantations are the gold standard assay to measure the regenerative capacity of blood stem cells. Aged HSCs engraft recipient mice less efficiently than young stem cells and show a myeloid differentiation skewing and reduced reconstitution of lymphoid cells (B and T cells). This phenotype is an intrinsic characteristic of aged blood stem cells, which is not improved by several rejuvenating interventions, like caloric restriction, physical exercise or parabiosis^39^ and largely account for the functional decline of the immune system in the elderly. Notably, aged HSCs sorted from CASIN mice reconstituted both primary and secondary recipients significantly better than aged HSC sorted from control mice (Fig. 4b, c). Moreover, B cell frequency was significantly higher in mice transplanted with aged HSCs from CASIN mice in both primary and secondary transplantations (Fig. 4b, c). Myeloid cell frequency was decreased compared to control only in primary recipients and only at 24 weeks after transplant (Fig. 4b, c). We did not detect differences in the BM compartment between samples, beside a significant decrease in the total BM engraftment in aged and CASIN primary transplanted mice and a decrease in LMPPs and an increase in HSCs only for CASIN recipients compared to young (Supplementary Fig. 5a, b). Altogether, we conclude that CASIN increases the regenerative potential and the lymphoid B cells differentiation capacity in peripheral blood of aged blood stem cells, while lymphoid-primed progenitors in BM didn’t show to increase in CASIN recipients compared to aged.

### CASIN treatment restores a youthful transcriptional heterogeneity in aged HSCs and improves connectivity across hematopoietic stem and progenitor cell clusters

**Fig. 5:**
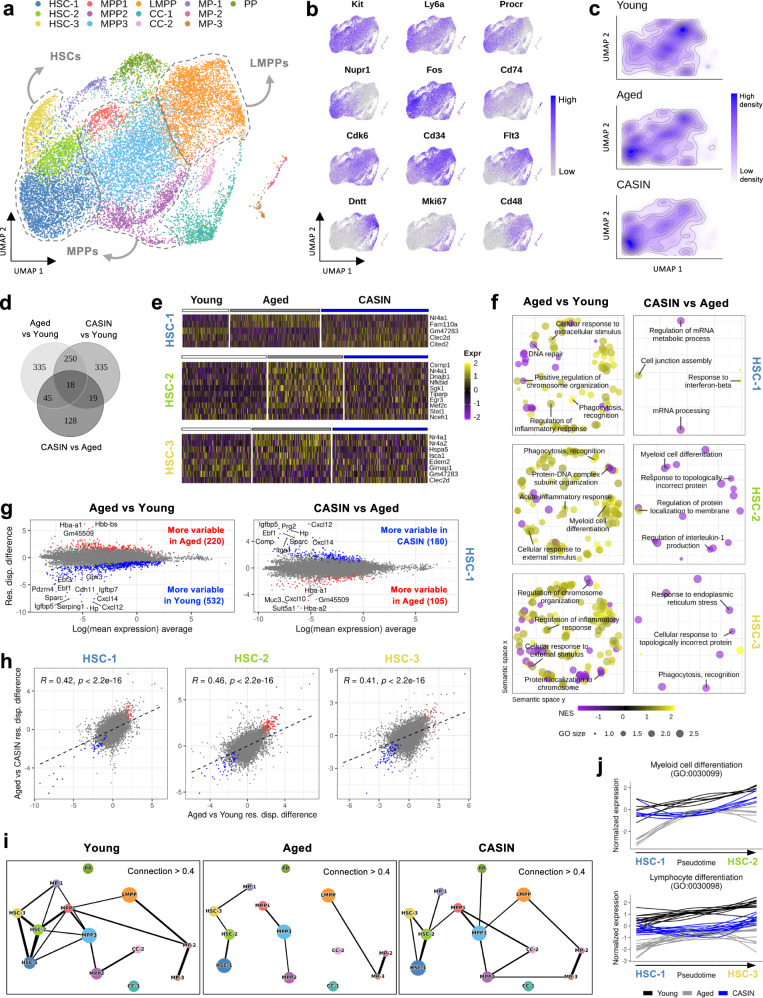
CASIN treatment restores a youthful transcriptional heterogeneity in aged HSCs and improves connectivity across hematopoietic stem and progenitor cell clusters. **a** UMAP with clusters obtained for LSK cells of young, aged and CASIN mice (defined in Supplementary dataset 2). **b** Expression levels of several marker genes. **c** Density in the UMAP for the young, aged and CASIN cells. **d** Venn diagram of the common DE genes in the three pairwise comparisons. **e** Single-cell expression levels of the top genes that are DE between CASIN and aged samples in clusters HSC-1, HSC-2, and HSC-3. **f** Gene Ontology (GO) terms obtained in the GSEA of the DE genes. NES: normalized enrichment score. GO size: size of the gene set. **g** Residual dispersion difference against average logarithmic mean expression for every gene for the HSC-1 cluster. Top 15 significantly differentially variable genes shown. **h** Spearman correlation of the residual dispersion difference of aged over young and aged over CASIN. Genes that are more (red) or less (blue) variable in both comparisons are highlighted. **i** PAGA plots representing the connections between clusters for young, aged and CASIN cells. The thickness of the lines represents the strength of the connections. Only connection values >0.4 are represented. **j** Expression pattern over pseudotime of several genes (Supplementary Dataset 10) involved in the indicated GO processes. Data are provided in Supplementary Dataset 1–10.

To further understand the improved phenotype of aged HSCs after in vivo CASIN treatment, we performed scRNA-seq of Lineage^−^(Cd11b^−^, Ter119^−^, Cd8^−^, Cd5^−^, B220^−^, Gr1^−^), C-kit^+^, Sca1^+^ BM cells (LSKs), which include all hematopoietic stem and progenitor cells in the BM (Supplementary Fig. 6a). A total of 15,856 LSK cells were obtained after the quality control and filtering (Supplementary Dataset 1), which divided into 13 clusters by using Seurat’s functions^40^ (Fig. 5a). The cell clusters were annotated based on previously reported marker genes (Fig. 5a, b, Supplementary Fig. 6b–e, Supplementary Dataset 2, and “Methods”section). Overall, we identified 3 clusters of HSCs, which presented with slightly different transcriptional programs (more quiescent, HSC-1; more active, HSC-2; less regenerative, HSC-3; see “Methods” section), while being all three equally associated with a primitive HSC signature^41^.

Many previous studies and our own data (Supplementary Fig. 4c, d) showed a 2- to 5-fold increase in HSC number and frequency with age. We used compositional analysis with scCODA^42^ to determine if the scRNA-seq profiling detects a similar increase in the proportion of HSCs in the aged LSK compartment. Interestingly, only the HSC-1 cluster, which is characterized by the most dormant gene signature, showed a significant cell number increase in both the aged and CASIN groups compared to the young samples, while cell number in LMPPs cluster is not significantly reduced in both aged and CASIN samples (Fig. 5c, Supplementary Fig. 6f, and Supplementary Dataset 3). Hence, we concluded that only cluster HSC-1 expand with aging regardless of CASIN. Some previous studies reported also a characteristic decrease in the proportion of HSCs in G1/S phase in aged compared to young mice by scRNA-seq profiling^43^. Therefore, we assessed for changes in the proportion of the different cell cycle phases for the three HSC clusters. We detected a decrease of cells in G1/S in aged compared to young only for cluster HSC-2, which encompasses a minor fraction of total HSCs. Interestingly, we measured a significant increase in the proportion of cells in G0 in CASIN samples for all HSC clusters, supporting that CASIN treatment preserves quiescence of aged stem cells, a characteristic in general associated with higher regenerative capacity^44^ (Supplementary Fig. 6g and Supplementary Dataset 3).

Differences in HSCs’ gene expression at the transcriptional level between aged and young mice have been reported in previous studies^43,45^. To determine if CASIN treatment has any effect on gene expression, we analyzed the differentially expressed (DE) genes between aged and young samples and compared them with the DE genes between CASIN and aged samples for each cluster. We obtained a total of 648 DE genes between aged and young samples and 210 DE genes between CASIN and aged samples (|log2FC|> 0.25 and Tippett’s combined p-value < 0.05 for Wilcoxon Rank Sum test; Fig. 5d, e, Supplementary Fig. 7, and Supplementary Dataset 4). Focusing on the DE genes between CASIN and aged control mice that revert to youthful level, *Nr4a1* and *Nr4a2* were downregulated in eight and four of our clusters respectively, including all HSC clusters (Fig. 5e, Supplementary Fig. 7, and Supplementary Dataset 4). These transcription factors and their activity is not regulated by ligand binding and are thus regulated via their expression levels and have been described to be involved in proliferation/quiescence and inflammation ^46,47^. *Dnajb1*, *Hspa5,* and *Edem2*, which are involved in the folding and assembly of proteins and the degradation of misfolded proteins in the endoplasmic reticulum and are activated under conditions of stress, were also downregulated after CASIN treatment in clusters HSC-2 and HSC-3 (Fig. 5e, Supplementary Fig. 7, and Supplementary Dataset 4). Gene Set Enrichment Analysis (GSEA) detected an upregulation of many pathways (for example inflammation, phagocytosis, myeloid differentiation) in all HSC clusters upon aging and it showed the downregulation of some specific pathways after CASIN, like myeloid differentiation, IL-1 production, phagocytosis, and mRNA processing. Notably few pathways were upregulated: the response to interferon-β and cell junction assembly in cluster HSC-1 and the regulation of protein localization in cluster HSC-2 (Fig. 5f and Supplementary Dataset 5). Overall, these results reveal selected changes in the transcriptome of HSCs in CASIN treated mice that are indicative of a downregulation in stress response and myeloid differentiation and of changes in the regulation of protein folding and localization, inflammatory response, and phagocytosis. However, GSEA results show that the transcriptional changes induced by CASIN are overall subtle compared to the transcriptional difference between young and old cells. Given the clear functional improvement, it might be possible that these discreet selected transcriptional changes are enough to increase the regenerative potential of HSCs. We can’t exclude broader epigenetic or transcriptional remodeling not yet detectable so early after the end of the treatment.

Next, we interrogated our dataset for HSC heterogeneity, since with aging hematopoietic clonality increases and HSC might become more homogeneous. Therefore, we tested the three HSC clusters for differential transcriptional variability between the conditions according to BASiCS^48^, using a regression model to improve the estimation of gene expression dispersion^49^. We identified a total of 1659 and 903 genes with differential variability in one or more HSC clusters between young and aged samples and between CASIN and aged samples, respectively (absolute residual dispersion difference >0.5 and posterior probability’s expected FDR < 0.05; Supplementary Dataset 6). By calculating the difference in the residual dispersions obtained for the aged and the young cells, we determined that, in all clusters, the number of genes becoming less variable with age (negative difference) was higher than the number of genes with increased variability (positive difference; 70.7%, 58.9%, and 77% showed a negative difference of the total number of genes with significant differences in variability for HSC-1, HSC-2, and HSC-3, respectively; Fig. 5g, Supplementary Fig. 8a, and Supplementary Dataset 6). These results indicate a lower transcriptional heterogeneity in aged HSCs. Focusing on the genes that showed differential variability after CASIN, we measured that for most of these genes (96.2%, 94.4%, and 96.3% for HSC-1, HSC-2, and HSC-3, respectively; Supplementary Fig. 8b) CASIN reverted the effects of aging on transcriptional variability. To further confirm this, we calculated the correlation between the residual dispersion differences in aged over young and in aged over CASIN, obtaining a significantly positive correlation in all the HSC clusters (*R* = 0.42, 0.46, and 0.41; Fig. 5h). The correlation between aged over young and CASIN over young is also positive (*R* = 0.6, 0.5, and 0.57 in each cluster; Supplementary Fig. 8c), indicating that the variability levels in the CASIN group are in a midpoint between the aged and the young levels. Overall, this data indicates a clear effect of CASIN on the transcriptional variability of aged HSCs increasing it to resemble the higher transcriptional heterogeneity of the young samples.

Further, we wondered if HSC priming and differentiation capacity might be affected by CASIN treatment^50^. Therefore, we focused on assessing the relative gene expression trajectories of the different HSC/multipotent progenitors (MPP) clusters. To this end, we used Partition-based Graph Abstraction (PAGA) ^51^, which quantifies the transcriptional relationship between cells and measures overall connectivity between clusters. The stronger the connection between clusters, the higher the possibility of cells to progress from one cluster to the other, which can serve as a surrogate transcriptome-based estimation of the differentiation capacity of HSCs to MPPs. We observed a general and strong decrease in the connections between the clusters in the aged samples compared to the young (Fig. 5i and Supplementary Dataset 7). While in young samples clusters were overall well connected to each other, with aging the connectivity across HSC, MPP and more differentiated clusters were broken, which suggests a reduced differentiation capacity of aged hematopoietic cells. Interestingly, in CASIN samples, clusters showed a general increase in the connection values compared to the aged group (Fig. 5i and Supplementary Dataset 7), with the connection values among HSCs increasing between 14 and 45% and the connections between HSC-1 and progenitors increasing of 115%. In conclusion, CASIN partially restored the relationships between cell clusters, suggesting an improved capacity of HSCs to commit and differentiate, which represents a critical aspect underscoring the regenerative capacity of blood stem cells.

Next, we identified the genes driving the changes in the connection values after CASIN treatment by selecting those with significantly different patterns of expression along trajectories in aged and CASIN samples using TradeSeq^52^ (Supplementary Dataset 8) followed by GSEA (Supplementary Dataset 9). Data revealed the expression of genes involved in myeloid cell differentiation pathways to be increased in the transitions from HSC-1 to HSC-2, HSC-1 to MPP1, and HSC-2 to MPP1 (Fig. 5j, Supplementary Fig. 8d and Supplementary Dataset 10), with CASIN samples resulting in a youthful expression pattern for these pathways. Similar results were observed for lymphocyte differentiation and B cell activation in the transitions from HSC-1 to HSC-2, HSC-3 and MPP1 (Fig. 5j, Supplementary Fig. 8d, and Supplementary Dataset 10). Moreover, in the transitions from HSC-1 to HSC-2 and from HSC-1 to MPP1 we also detected a decrease in the expression pattern of genes associated to TGF-β signaling in young and in CASIN samples, while TGF-β signaling remained sustained in the aged clusters (Supplementary Fig. 8d and Supplementary Dataset 10). These data suggest that CASIN might improve the differentiation capacity of aged HSCs by better coordinating gene expression changes across cell clusters with respect to both myeloid and lymphoid priming as well as by downregulating TGF-β signaling.

### Blood stem cells from CASIN mice extend median and maximum lifespan of aged immunocompromised mice

**Fig. 6:**
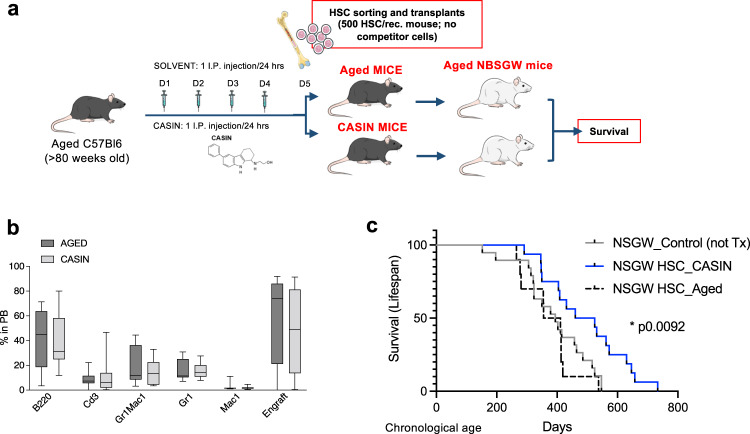
Blood stem cells from CASIN mice extend murine median and maximum lifespan. **a** Experimental design for non-competitive transplants in not-preconditioned immunocompromised aged mice. 500 sorted HSCs were transplanted without competitor cells in aged NBGSW mice. **b** Flow cytometry quantification of peripheral blood profile at 4 months after transplantation of aged NBSGW recipient mice. (Aged, *n* = 7; CASIN, *n* = 9). Columns are mean ±SE. **c** Survival after Tx and overall lifespan of NBGSW mice. (Aged, *n* = 10; CASIN, *n* = 16; Not transplanted, *n* = 19). Survival analysis with Mantel Cox test. Source data are provided as a Source Data file. Mouse cartoon: CreativeBucket:stock.adobe.com. Bone and cells cartoon: smart.servier.com.

Previously, we showed that systemic CASIN treatment extends median and maximum lifespan of aged mice^18^ and now we demonstrate a direct effect of this same treatment on aged HSCs. Since the hematopoietic system is the carrier for many rejuvenation factors, we wondered whether in vivo rejuvenated blood stem cells exert any effect on murine lifespan. To address this point, we decided to transplant aged HSCs harvested after systemic CASIN treatment into aged immunocompromised and *Kit*^*W-41J*^ mutant mice (NBSGW), which can be efficiently engrafted by congenic murine cells without irradiation^10^ (Fig. 6a). This experimental setting allows to use aged mice as recipients without stressing them with pre-conditioning treatments (for example irradiation or sub-lethal irradiation or parabiosis) that might reduce or alter their lifespan. We non-competitively transplanted more donor stem cells compared to the competitive transplantation setting used above (see Fig. 4a). This higher number of donor HSCs allowed on average equal high engraftment of recipients of both aged and CASIN mice, excluding that differences in engraftment levels or a low engraftment level would bias the survival outcome^53^ (Fig. 6b). Notably, aged NBSGW mice transplanted with HSCs from CASIN mice survived significantly longer than mice transplanted with aged control HSCs (Supplementary Fig. 8e). Importantly, while mice transplanted with aged control HSCs showed no changes in lifespan compared to the cohort of not transplanted mice, aged mice receiving HSCs from aged CASIN treated mice presented with a significant increase in median (+24.8%) and maximum (+34.0%) lifespan (Fig. 6c). Therefore, our data support that blood stem cells rejuvenated by CASIN treatment are sufficient to extend lifespan of aged immunocompromised mice.

## Discussion

The ability to restore or rejuvenate aged tissues by targeting endogenous stem cells is a central goal of regenerative medicine. However, systemic rejuvenation of aged stem cells remains a challenge and it is still unclear to what degree do stem cells contribute to overall organism health- and lifespan. Here, we show that a brief systemic treatment of aged mice with the Cdc42-activity inhibitor CASIN^17^ improves the regenerative potential of endogenous aged MuSCs and HSCs in vivo. We report that after CASIN treatment aged MuSCs divisional kinetic and myogenic capacity in vitro are enhanced and, after injuring the muscle in vivo with Ntx, tissue regeneration is improved. Supporting that the MuSC improvement after CASIN might contribute to extend mouse healthspan, CASIN mice performed better than aged control mice in endurance and strength tests in steady-state and also after Ntx damage. Moreover, we report on systemic CASIN affecting Cdc42 and tubulin polarity as well as H4K16ac epigenetic polarity in aged HSCs. The data on H4K16ac obtained by histological analyses of whole mount BM sections aligns to those previously reported on the DNA-methylation-based epigenetic clock^18^ and strongly support at least some traits of epigenetic rejuvenation in HSCs after systemic CASIN treatment. Furthermore, the histological analysis shows an intriguing effect of CASIN also on aged HSC localization, which after the treatment is closer to arteries and endosteum, like young blood stem cells. At the transcriptional level, stress response and inflammation constitute the major signaling pathways targeted by CASIN in vivo. Consistently, we have previously reported a significant reduction in the levels of inflammatory cytokines (IL1α, IL1β, and INFγ) in peripheral blood serum of aged mice after in vivo CASIN treatment^18^. These same cytokines were also shown by others to play critical roles in aging of the blood and other tissues^54,55^.

The hematopoietic system is the carrier for many rejuvenation factors, and this leaves open the possibility that rejuvenating aged HSCs might represent an effective strategy to improve aging of the whole organism. Here we show that upon transplantation rejuvenated blood stem cells are sufficient to increase murine lifespan of aged immunocompromised mice. Altogether these results raise critical considerations for refining the targets and goals of anti-aging strategies focusing on a possible central role of HSCs and of the hematopoietic system^56^. To note, previous data associated increased Cdc42 activity to aging in humans and in aged human HSCs, supporting the translational potential of these findings^12,57,58^.

These data, together with the recent data supporting an improved activity of aged intestinal and hair follicle stem cells after CASIN treatment^13,15^ support that the increased activity of Cdc42 with aging impairs the function of several somatic stem cells in different tissues^59^. Therefore, systemic treatment with CASIN can elicit distinct positive biological effects in vivo, which might depend on the doses and way of administration. Besides, recently Cdc42 activity has been shown to limit the lifespan of the budding yeast, hinting at a phylogenetically conserved mechanism of the Cdc42-polarity axis in affecting organism aging^6^.

Overall, our data supports that aged stem cells exert a relevant function in the decline of organism fitness with aging. Further, we provide proof-of-concept evidence that increasing the regenerative potential of endogenous aged somatic stem cells might indeed represent an important strategy for rejuvenating tissues and improving health- and lifespan in the elderly.

## Methods

### Mice

Young and aged C57BL/6 mice were obtained from the internal divisional stock (derived from mice obtained from both The Jackson Laboratory and Janvier), as well as from NIA/Charles River. The NBSGW mice (JAXStock No.026622) were maintained as homozygotes. Cdc42GAP mice were described previously^8^. All mice were housed in the animal barrier facility under pathogen-free conditions at the University of Ulm and at the Biomedical Research Institute of Bellvitge (IDIBELL). Throughout the manuscript, young C57BL/6 mice are between 10 and 16 weeks old and aged C57BL/6 mice are at least 80 weeks old. C57BL/6 mice were randomized for sex.

For the lifespan study NBSGW mice were randomized for sex and equal number of male and female mice were used across samples. The average lifespan of NBSGW mice in our hands is 56 weeks and therefore mice older than 32 weeks were considered “old”. Mice that didn’t show engraftment at any time point were excluded from the study. Mice that failed to recover from blood sampling and mice that died due to laboratory errors were excluded. Mice that needed to be euthanized because they were scored as “weak and about to die” according to our approved animal license protocol for evaluating mouse health status remained part of the dataset. Allocation to control or treated group was done randomly (5–10 mice each experimental group in two different experimental batches).

Median lifespan, 95% confidence intervals and survival analysis were calculated by Prism GraphPad v9.3.1. All animals were maintained according to the recommendations of the European Convention for the Protection of Vertebrate Animals used for Experimental and other Scientific Purposes (ETS 123). Animals were housed in groups of up to 4 animals per cage in Macrolon Type II (long) cages with bedding and paper nesting material. The animals had access to food (V1124-3, ssniff®) and water *ad libitum*. Animals were kept at a day/night rhythm of 12/12 h throughout the entire experiment.

### Ethical compliance for mouse experiments

All mouse experiments were performed in compliance with the ethical regulations according to: (1) the German Law for Welfare of Laboratory Animals and were approved by the Institutional Review Board of the Ulm University, as well as by the state government of Baden‐Württemberg, Regierungspraesidium Tuebingen; (2) the Spanish Law for Animal Protection and Welfare Code and were previously approved in the project AR18008/10399 by IDIBELL’s Ethical Committee for Animal Experimentation (CEEA-IDIBELL) as well as by Generalitat of Catalunya.

### CASIN treatment in vivo

All C57Bl6 young and aged mice were subjected to health check-up and weighed prior to enter the study. We didn’t stratify the mice based on sex and equal number of male and female mice were used in each group. After this initial assessment, mice were randomized to either the CASIN or the control group. CASIN injections were perfomed i.p. for 4 days, one injection every 24 h at a dose of 25 mg/kg in cyclodextrin (H5784, Sigma Aldrich). Young and aged control mice were injected with equivalent amount of cyclodextrin only. CASIN was prepared fresh before injection by dissolving the drug directly in beta-cyclodextrin solution. I.p. injections were done in the morning every 24 h for 4 consecutive days. The same stock of CASIN was used for the whole study. CASIN was kindly provided by Dr. Yi Zheng as lyophilized powder and belonged to the same production batches characterized in ref. ^17^.

### Western blot and Cdc42-GTPase effector domain pull-down assays

For western blot and pull-down assays, hindlimb muscles were minced with a scalpel and tissue pieces were moved into MACS M-tubes (Milteny Biotech). Tissue pieces were centrifuged (520 × *g* 5 min 4 °C), weighted and then for each 100–200 mg of tissue 2 mL of cold protein lysis buffer was added. The protein lysis buffer was Mg^2+^ lysis/wash buffer (Upstate cell signaling solutions) containing 10% glycerol, 25 mM sodium fluoride, 1 mM sodium orthovanadate and a protease inhibitor cocktail (Roche Diagnostics). Tissues were lysed by running the gentleMACS Protein Program according to the manufacturer protocol (Milteny Biotech). Afterwards, protein lysates were left on ice for 1 h and then centrifuged at 17,000 × *g* for 10 min 4 C. The supernatant was collected and stored at −20 °C or used immediately for pull down.

Relative levels of GTP-bound Cdc42 were determined by an effector pull-down assay. Briefly, samples were incubated with PAK-1 binding domain/agarose beads and bound (activated) as well as unbound (non-activated) Cdc42 fractions were probed by immunoblotting with an anti-Cdc42 antibody (Millipore, rabbit polyclonal). Activated protein was normalized to total protein and/or β-actin (Sigma) and the relative amount was quantified by densitometry. Representative uncropped blots are in Source Data file. More information on the antibodies used is provided in the Source Data file.

### Muscle flow cytometry and single cell sorting

Muscles from both hindlimbs (TA, EDL, gastrocnemius, plantaris, soleus) and/or Notexin-injured TA muscles from young, aged, and CASIN mice where indicated were harvested. Following mincing and enzymatic digestion with Skeletal Muscle Dissociation Kit mouse and rat (130-098-305, Miltenyi Biotec) and gentleMACS octo dissociator (130-095-937, Miltenyi Biotec), the digested tissue was centrifuged at 520 × *g* at 10 min. The pellet was resuspended with DMEM (high glucose, pyruvate, 41966029, Invitrogen) +10%FBS (F2442, Sigma Aldrich) and filtered through MACS Smartstrainers 70 µm (130-098-462, Miltenyi Biotec) and 30 µm (130-098-458, Miltenyi Biotec). The cell suspension was stained with lineage negative antibodies for 20 min at 4 °C. Incubation with FcBlock (CD16/CD32, Clone 93, 14-0161-81, Invitrogen) was done for 15 min at 4 °C. Finally, stainings with the antibodies, Brilliant Violet 421- conjugated CD184 and APC- conjugated CD29 was performed for 60 min at 4 °C. Single cells were sorted directly in Terasaki plates for further analyses or subjected to flow cytometry analyses. Prior to sorting, the Terasaki plates (72 well with lid 83.3 × 58 × 10 mm, 654102, Greiner Bio-One) were coated with 5 ul/well of PBS + Ca/Mg (L 1815, Biochrom) containing collagen (1 mg/ml, 9007-34-5, Sigma Aldrich) and laminin (50 μg/ml, Cat# 23017-015, Invitrogen) for at least one hour at 37 °C. On top of the coating media, 15 μl/well of culture medium Ham’s F-10 Nutrient Mix (11550-043, Thermo Fisher Scientific) along with 20% Horse serum (H1270, Sigma Aldrich), 1% Pen/Strep (P11-010, PAA laboratories) and 2.5 ng/ml of recombinant murine FGF basic (450-33, Peprotech) for further culture. More information on the antibodies used is provided in the Source Data file.

### CASIN treatment ex vivo

CASIN (10 mM in DMSO, M60040-2, Xcessbio) at a concentration of 0.5 µM and 5 µM in culture medium was used to treat isolated myofibers and sorted MuSCs from aged mice overnight for 24 h and 48 h respectively.

### Growth curve and clonal division analysis

Single sorted MuSCs from young and aged mice in Terasaki plates were analyzed for their growth kinetics and clonal divisions at different time-points, 2 h, 17 h, 24 h, 48 h and day 6 where indicated.

### Immunostainings of skeletal muscle tissue samples

Control and injured TA muscles were harvested and processed from young, aged, and CASIN mice as described in ref. ^60^. In brief, the harvested TA muscles were fixed in 4% for 2 h at 4 °C, washed twice in PBS (P04-36500, PAN-Biotech). Later, they were then transferred into 20% sucrose (S0389, Sigma-Aldrich) in PBS overnight at 4 °C followed by 3–4 h in 30% sucrose. The TA muscles were placed in Tissue Tek Cryomold 25 mm × 20 mm × 5 mm (4557, Sakura) and OCT compound (4583, Sakura) and immediately frozen in liquid nitrogen-cooled Iso- pentane (M32631, Sigma-Aldrich) and/or dry ice. In all, 8 μm cryosections were cut with a cryostat (HM560M, Thermo Fischer Daigonstic GmbH) on superfrost slides (10417002, Thermo-Scientific). For stainings, the cryosections were thawed at RT, washed once with PBS. Antigen Retrieval with Citrate Buffer pH 6.0 (C9999, Sigma-Aldrich) was performed. Fixation with 4% PFA for 10 mins, permeabilization with 0.5% Triton X 100 for 8 min and blocking with 20% Normal Goat Serum (X090710-8, Dako) at RT. Primary antibodies were diluted in PBS O/N at 4 C, washed twice with PBS. Secondary antibodies were diluted in PBS for 1 hr at RT, washed again twice with PBS and mounted using Fluorescence Mounting Medium (S3023, Dako). More information on the antibodies used is provided in the Source Data file.

### FACS staining and sorting of HSC

Mononuclear BM cells were isolated by low-density centrifugation (Histopaque 1083, Sigma) and stained with a cocktail of biotinylated lineage antibodies. Biotinylated antibodies used for lineage staining were all rat anti-mouse antibodies: anti-CD11b (clone M1/70), anti-B220 (clone RA3-6B2), anti-CD5 (clone 53-7.3), anti-Gr-1 (clone RB6-8C5), anti-Ter119 and anti-CD8a (Clone 53-6.7) (all from eBioscience). After lineage depletion by magnetic separation (Dynalbeads, Invitrogen), cells were stained with anti-Sca-1 (clone D7) (eBioscience), anti-c-kit (clone 2B8) (eBioscience), anti-CD34 (clone RAM34) (eBioscience), anti-CD127 (clone A7R34) (eBioscience), anti-Flk-2 (clone A2F10) (eBioscience), and Streptavidin (eBioscience). Early haematopoiesis FACS analysis data were plotted as percentage of long-term haematopoietic stem cells (LT-HSCs, gated as LSK CD34^−/low^Flk2^−^), short-term haematopoietic stem cells (ST-HSCs, gated as LSK CD34^+^Flk2^−^), and lymphoid-primed multipotent progenitors (LMPPs, gated as LSK CD34^+^Flk2^+^) distribution among LSKs (Lin^neg^c-kit^+^sca-1^+^ cells). In order to isolate HSCs, lineage depletion was performed to enrich for lineage negative cells. Lineage negative cells were then stained as aforementioned and sorted using a BD FACS Aria III (BD Bioscience). More information on the antibodies used is provided in the Source Data file.

### Immunofluorescence staining of sorted HSCs

FACS-sorted HSCs were immunostained according to ref. ^9^. Briefly, freshly sorted HSCs were seeded on fibronectin-coated glass coverslips. For polarity staining, HSCs were incubated 12–16 h in HBSS + 10%FBS at 37 °C, 5%CO_2_, 3%O_2_. Cells were fixed with BD Cytofix Fixation Buffer (BD Biosciences). After fixation cells were gently washed with PBS, permeabilized with 0.2% Triton X-100 (Sigma) in PBS for 20 min and blocked with 10% Donkey Serum (Sigma) for 30 min. Primary and secondary antibodies incubations were performed for 1 h at room temperature. Samples were imaged with an AxioObserver Z1 microscope (Zeiss) equipped with a 63X PH objective. As for polarity scoring, the localization of each single stained protein was considered polarized when a clear asymmetric distribution was visible by drawing a line across the middle of the cell or the nucleus. On average, a total of 20–50 HSCs were singularly analyzed per sample. Data are plotted as percentage of the total number of cells scored per sample. More information on the antibodies used is provided in the Source Data file.

### HSC competitive transplantation

For competitive HSC transplantation, young and aged±CASIN C57BL/6 mice (Ly5.2^+^) were used as donors. 200 HSCs were sorted into Terasaki plates and maintained for 16 h in HBSS + 10%FBS in a water-jacketed incubator at 37 °C, 5%CO_2_, 3%O_2_. Stem cells were then mixed with 3×10^5^ BM cells from young (2–4-month-old) BoyJ competitor mice (Ly5.1^+^) and transplanted into BoyJ recipient mice (Ly5.1^+^). Peripheral blood chimerism was determined by FACS analysis every 8 weeks up to 24 weeks post primary transplants. The transplantation experiment was performed three times with a cohort of 5 recipient mice per group each transplant. In general, transplanted mice were regarded as engrafted when PB chimerism was higher or equal to 1.0% and contribution was detected in all lineages. Secondary transplants were performed using 10^6^ total BM cells from primary transplanted mice injected i.v. directly into irradiated BoyJ recipient mice (Ly5.1^+^).

### HSC non-competitive transplantation in NBSGW mice

For non-competitive HSC transplantation in immunocompromised mice, aged (>34-week-old) NBSGW mice (Ly5.2^+^) were used as recipients. In all, 500 HSCs were FACS sorted from aged C57BL/6 mice expressing constitutive red fluorescenct protein (RFP)^61^ (RFP^+^Ly5.2^+^) into Terasaki plates and maintained for 16 h in HBSS + 10%FBS in a water-jacketed incubator at 37 °C, 5%CO_2_, 3%O_2_. Stem cells were then transplanted into recipient mice. PB chimerism was determined by FACS analysis for RFP^+^ cells every 4 weeks up to 24 weeks post-transplant. The transplantation experiment was performed two times with a cohort of 5–10 recipient mice per group each transplant. In general, transplanted mice were regarded as engrafted when PB chimerism was higher or equal to 0.5% and contribution was detected in all lineages.

### Flow cytometry of PB

PB cell immunostaining was performed according to standard procedures and samples were analyzed on a LSRII flow cytometer (BD Biosciences). For PB lineage analysis the antibodies used were all from eBioscience: anti-CD3ε (clone 145-2C11), anti-B220 (clone RA3–6B2), anti-Mac-1 (clone M1/70), and anti-Gr-1 (clone RC57BL/6-8C5). Lineage FACS analysis data are plotted as the percentage of B220^+^, CD3^+^, and Myeloid (Gr-1^+^, Mac-1^+^, and Gr-1^+^ Mac-1^+^) cells among total white blood cells. WBC, RBC, Ly, NE, Mo counts were generated by using the hemocytometer Hemavet 950, DREW SCIENTIFIC Inc, (FL 33014), USA. More information on the antibodies used is provided in the Source Data file.

### Myofibers Immunostainings

After 24 h in culture, Myofibers were stained according to ref. ^60^. Myofibers were washed with PBS twice and fixed with 4% PFA for 8 min at RT, followed by 2 times PBS wash. Permeabilization with 0.5% Triton X100 (T 8787, Sigma-Aldrich) for 8 min at RT, blocking with 20% Horseserum in PBS for 1 h at RT. Primary antibodies were diluted in PBS O/N at 4 C. Myofibers were washed twice with PBS + 0.05% Triton × 100. Secondary antibodies were diluted in PBS for 1 hr at RT, washed again twice with PBS + 0.05% Triton × 100 and mounted using Fluorescence Mounting Medium (S3023, Dako). More information on the antibodies used is provided in the Source Data file.

### Endurance test

The endurance of the mice was tested with Rotarod instrument (Ugo Basile, Gemonio, Italy, model 47600), having a rod of 3 cm in diameter and five lanes with a width of 5 cm each. Accelerated (Ramp Mode) rotarod tests (5–20 rpm with 120 secs as ramp time) were performed for maximum of 5 min (300 s) to evaluate the muscle endurance strength. Latency of fall is measured when the mouse falls off the rod. Prior to the analysis, the mice were trained for 3–5 min. Three measurements were performed which were averaged later for each mouse. The maximum time for the endurance test was 5 min (300 s) for each measurement and 10 min of resting period was given after each measurement.

### Notexin induced injury (regeneration assay)

For examining the effect of CASIN on regeneration in aged mice, firstly, the mice were anesthetized with 100 mg/kg bodyweight of Ketanest (Ampulle 25 mg/ml, 37087, Pfizer), Rompun 2% (Xylazin, Bayer) in 0.9% NaCl (Fresenius Kabi Deustchland) 15 μl of Notexin (100 µg, L8104, Latoxan) was injected in either one (regeneration) or both (endurance) TAs. The contralateral TA muscle was used as the uninjured control. Mice were injected subcutaneously with an analgesic 1 ml Temgesic® (Buprenorphin 0,3 mg/ml, 700248 (PZN)00345928). After 4 days of injury, the TA muscles were harvested and processed for further analyses.

### Myofiber isolation

Myofibers from EDL (Extensor Digitorum Longus) of young and aged mice were isolated in accordance with ref. ^62^. The isolated myofibers digested using Collagense Type I (Cat# SCR103, Sigma-Aldrich) were grown cultured in 8-well Chamber Slide (177445PK, Thermo Fisher Scientific) coated with Corning® Matrigel® (734-0272, VWR) in DMEM,1% Pen/Strep, 1% Chick embryo extract (092850145, MP Biomedicals) at 37 °C. After 24 h in culture with and without CASIN, the myofibers were fixed for immunostainings.

### iFAST3D whole mount immunofluorescence staining of BM

Whole mount histology protocol was performed as previously described^34,37^. Vasculature was stained by intravenous injection of APC-anti-CD31 (clone MEC13.3, BioLegend) and Alexa Fluor 647-anti-CD144 (clone BV13, BioLegend) antibodies. Subsequently, bones were harvested after post-mortem heart perfusion with 4% paraformaldehyde (PFA) in phosphate-buffered saline (PBS). Bones were post-fixed in 4% PFA/PBS-solution for 24 h at 4 °C and they were embedded in an optimum cutting temperature compound (OCT, Tissue-Tek) and were snap frozen in liquid nitrogen and stored at −80 °C. Bones were shaved along the longitudinal axis on a cryostat (Leica CM3050S) until the BM cavity was exposed. The bones were collected for the staining step by melting the optimum cutting temperature compound in PBS at room temperature. Shaved bones were fixed again in 4% PFA/PBS at room temperature for 30 min. Shaved bones were blocked and permeabilized with buffer containing 20% donkey serum and 0.5% Triton X-100 in PBS with 1% Penicillin/Streptomycin solution (P/S). Immunostaing of the BM cavity was performed by incubatin the shaved bones with a fluorescently labelled antibody PE-anti-CD150 (clone TC15-12F12.2, BioLegend), Biotin-labelled primary antibodies anti-CD41 (clone MWReg30), anti-CD48 (clone HM48-1), anti-CD11b (clone M1/70), anti-B220 (clone RA3-6B2), anti-CD5 (clone 53–7.3) anti-Gr-1 (clone RB6-8C5), anti-Ter-119, and anti-CD8a (Clone 53–6.7) (eBioscience) and anti-H4K16ac (07-329, Millipore) for 1–3 days at 4 °C and stained with Streptavidin-eFluor 450 (eBioscience) and AF488 anti-rabbit antibody for 1.5 h at room temperature. Shaved bones were incubated with FITC-anti-Ki-67 (clone SolA15, ebioscience) for 3 h at room temperature for proliferation analysis. More information on the antibodies used is provided in the Source Data file.

### iFAST3D whole mount histology image acquisition and analyses

Immunostained images from cross-sections, sorted HSCs and myofibers were captured and analyzed using Light microscope (CKX31) for growth curve and clonal divisions, Epifluorescence microscope with ×10 and ×40 (objectives Zeiss AxioObserver Z1 microscope), Confocal microscope (LSM710, Zeiss) equipped with 20x and 63x. ImageJ and its plugins (National Institutes of health, USA) were used for processing images, measuring cross-sectional areas (CSA) and Pax7^+^, MyoD^+^ cells. For the iFAST3D whole mount histological analyses^34^, shaved bones were imaged with Zeiss LSM 880 confocal microscope and analyzed with the image analysis software Volocity (v7.0, Quorum Technologies). HSCs were manually labeled using the point tool of the Volocity software after their identification based on the following objective criteria: a diameter from 5–6 to 10-12 µm, the exclusive expression of CD150 after cleaning the signal from its background (remove noise and contrast/enhancement functions of the Volocity software) and no overlap with any signal derived from the exclusion markers staining nor from the one associated with vasculature, evaluated after removing the background signal (remove noise and/or contrast/enhancement functions of the Volocity software). Subsequently, H4K16ac polarity and Ki67 staining were evaluated in the identified HSCs and HSCs were differentially labeled using the point tool of the Volocity software accordingly on displaying a polar or an apolar distribution of the H4K16ac or being positive or negative for Ki67 expression. The nearest distances from the identified HSCs to multiple niche cell types and structures were measured. The definition of arteriole includes arterial and arteriolar cell. Arterioles and sinusoids were distinguished by morphology and orientation. The minimum distance between HSCs and sinusoids and arterioles was calculated from the centroid of the identified HSC to the edge of the nearest vessel type by using the line tool of the Volocity software. The minimum distance from the endosteum was calculated from the centroid of the identified HSC to the edge of the bone observed in bright field in touch with the immunostained BM by using the line tool option of the Volocity software. Proximity evaluation was performed considering: HSC in proximity to arteries or sinusoids if the minimum distance between the HSC and the vessel calculated using the line tool of the Volocity software was <10 µm and in proximity of the endosteum if the minimum distance between the HSC and the bone surface calculated using the line too of the Volocity software was <50 µm. Percentage of HSCs in proximity of the defined structures and distance measure were subsequently used for the analysis.

### Statistical analysis

For statistical analysis GraphPad Prism version 9.3.1 was used. Details about the number of biological replicates and statistical tests applied are included in the figure legends.

### Single-cell RNA sequencing of LSK cells

#### Quality control and filtering of the count matrices

Sequenced reads were mapped to the reference genome (GRCm38/mm10, annotation Ensembl 93, July 2018), filtered, and counted using Cell Ranger software v3.1.0 (10x Genomics).

Count matrices were pre-processed using R v4.0.2 and Seurat package v4.0.2^40^. Genes expressed in <3 cells were discarded. Cells with >10% of mitochondrial RNA, >6000 genes, <1000 UMI counts, or >40,000 UMI counts were also discarded. We further discarded cells that deviate from the regression line defined by the number of genes and number of counts in logarithmic scale with a residual error >0.4. We obtained a total of 15,856 cells and 19,517 genes to continue with the analysis. More information on the number of cells, genes, and UMI counts for every sample can be found at Supplementary Dataset 1.

### Cell cycle scoring

The genes defined by Macosko et al.^63^ associated to G1/S, S, G2/M, and M phases of the cell cycle, based on the cell cycle genes previously defined by Whitfield et al.^64^ in HeLa cells, were used to score each cell for the average expression of each set of genes. According to Whitfield et al., G1/S genes were characterized for being involved in DNA replication, packaging, and repair; S genes were involved in nucleotide metabolism and histones; G2/M genes produced tubulin and mitotic spindle assembly proteins; and M genes participated in mitotic surveillance. Before scoring, gene sets were filtered by calculating the Pearson correlation between the raw expression of each gene and the average expression of all the genes associated to the same phase and keeping only the genes with correlations higher than 0.3, as defined by Macosko et al.^63^. The scores for each cell cycle phase were calculated using the AddModuleScore function of Seurat and the log-normalized data and a cell cycle phase was assigned to each cell according to the highest score. G0 phase was assigned when all scores were negative.

### Sample integration, dimensionality reduction, and clustering

Gene counts for each sample were separately normalized using Seurat’s SCTransform function^65^, regressing out the effect of the number of genes, the number of UMI counts, the percentage of mitochondrial RNA and the scores for the cell cycle phases in every cell and returning 3000 variable genes for each sample. Integration was performed following Seurat’s integration workflow, using 3000 integration features and canonical correlation analysis with 30 dimensions. We generated the UMAP using the integrated dataset, 35 principal components (PCs), and 500 epochs. Clustering was performed with Seurat’s FindNeighbors and FindClusters functions, using 35 PCs, Louvain algorithm and a resolution of 0.4, chosen after evaluating several resolutions with the clustree package v0.4.3^66^.

### Cluster annotation

To annotate the clusters, we looked for their marker genes using the FindConservedMarkers function of Seurat, with the two sequencing batches as the grouping variable and using the log-normalized gene counts and the Wilcoxon Rank Sum test. We only tested those genes expressed at least in 10% of the cells in either of the two populations and we considered them as markers if they had a positive log2 fold change (log2FC) higher than 0.25 and a maximum p-value lower than 0.01. We compared the obtained marker genes with the genes described in the literature to assign a cell type to each cluster.

Genes associated to hematopoietic stem cells (HSC), like *Procr*^67^, *Fgd5*^68^, *Sult1a1*^68^, and *Nupr1*^69^ were mainly expressed in the three clusters at the left side of the UMAP, consequently labeled as HSC-1, HSC-2, and HSC-3 (Fig. 5a, b and Supplementary Dataset 2). The expression of these genes is especially high in HSC-1, which also shows a very low expression of *Cdk6* and *Cd34* and higher expression of *Meg3* (Fig. 5b, Supplementary Fig. 6b, and Supplementary Dataset 2), a signature that was described to be characteristic of dormant HSCs^70^. HSC-2 cluster is characterized by a high expression of the AP-1 transcription factor complex genes, like *Fos* and *Jun* (Fig. 5b, Supplementary Fig. 6b, and Supplementary Dataset 2), that are known to act in response to stress and whose high expression has been suggested to be associated with an increased capacity of HSCs for differentiation and commitment^71^. Interestingly, HSC-3 cluster is enriched in *Cd74* and other Major Histocompatibility Complex II (MHC II) genes, like *H2-Aa*, *H2-Eb1* and *H2-Ab1* (Fig. 5b, Supplementary Fig. 6b, and Supplementary Dataset 2). A recent study by Becker-Herman et al.^72^ showed that an absence of *Cd74* leads to an increased HSC repopulation and self-renewal capacity, suggesting that this cluster might group the less repopulating HSCs. To further confirm the annotation of these HSC clusters, we calculated Jaccard indexes comparing our markers with the signatures defined by Pei et al.^41^ for differentiation-inactive and multilineage HSCs. We used the the matchSCore2 package^73^ to calculate for every cluster the Jaccard similarity index comparing the 200 marker genes with higher log2FC (including genes with a Tippett’s combined p-value lower than 0.05) with the genes in the signatures. For all HSC clusters, we obtained positive indexes only for the “differentiation-inactive” signature and zeros for the “multilineage” one (Supplementary Figure 6c).

The central clusters in the UMAP were labeled as multipotent progenitors (MPP) because they present a higher expression of *Cd34* compared to HSCs, indicating a more differentiated state. They were assigned to the different subtypes of MPPs according to the expression of the marker genes *Cd150*, *Cd34*, *Cd48,* and *Ftl3*^67^ (Supplementary Fig. 6d). MPP1 (Cd150^+^Cd34^+^Cd48^−^Flt3^−^) is a cell type supposed to give rise to more lineage-biased MPPs. This cluster also presents a high expression of several differentiation-associated lncRNAs like *Malat1* and *Neat1*^74^ (Supplementary Fig. 6b and Supplementary Dataset 2). MPP2 (Cd150^+^Cd34^+^Cd48^+^Flt3^−^) is characterized by the expression of *Itga2b* and *Pf4* (Supplementary Fig. 6b and Supplementary Dataset 2), which are markers of megakaryocytes^75^ and^76^, supporting that this might be a megakaryocyte-biased cluster. The cluster MPP3 (Cd150^−^Cd34^+^Cd48^+^Flt3^−^) is expected to be biased towards the granulocyte/macrophage fate, although it shows very few specific marker genes (Supplementary Fig. 6b and Supplementary Dataset 2). Finally, the lymphoid multipotent progenitor (LMPP) cluster (Cd150^−^Cd34^+^Cd48^+^Flt3^+^) presents a high expression of *Dntt* and *Satb1* known to be characteristic of lymphoid biased progenitors^77^ (Supplementary Fig. 6b and Supplementary Dataset 2).

The rest of the clusters were annotated as cycling cells (CC), for showing high expression of cell cycle genes (Supplementary Fig. 6b and Supplementary Dataset 2) and a large proportion of cells in G2/M and M phases (Supplementary Fig. 6e); myeloid progenitors (MP), expressing genes like *Rsad2*^78^, *Ifit1*^79^, *Lgals1*^80^, *Irf8*^81^, *Elane*^82^, *Ctsg*^82^ and *Mpo*^82^ (Supplementary Fig. 6b and Supplementary Dataset 2); and other primed progenitors (PP) that did not express clear marker genes. A summary of the main marker genes, gates and references used for annotation can be found in Supplementary Dataset 2.

### Cluster proportions and compositional analysis

Cluster proportions were calculated using R and represented using the ggplot2 package v3.3.3. Python v3.8.10 and the scCODA package v0.1.2^42^ were used to assess the statistical credibility of the differences in cluster cell number between conditions. The model was run 10 times with young condition as the reference group and 10 times with aged condition as the reference group. In all runs, MP-1 was used as the reference cluster, for being one of the clusters showing less variability in its proportion between the samples. Those clusters for which a non-zero effect parameter (EP) was obtained in >6 runs were considered to have credible differences. A similar procedure was followed to assess the statistical credibility of the differences in the cell cycle phases cell number between conditions for clusters HSC-1, HSC-2, and HSC-3. For each of these clusters, the model was run 10 times with young and 10 times with aged condition as the reference group. The cell cycle phase showing less variability in its proportion between the samples was used as the reference phase (M for HSC-1 and HSC-3 and S for HSC-2). As before, if a non-zero EP was obtained in >6 runs, the differences in cell number for that cell cycle phase were considered credible.

### Differential expression analysis

For each cluster, differentially expressed genes in the three pairwise comparisons between the conditions were found using the FindConservedMarkers function of Seurat on the log-normalized gene counts. Only genes expressed in >10% of the cells in eiShather of the two cell populations were tested. Genes were considered differentially expressed between two conditions if they had an absolute log2FC higher than 0.25 in the two sequencing batches and a Tippett’s combined *p*-value <0.05 for Wilcoxon rank-sum test. Plots were generated using the R packages ggplot2 v3.3.3, patchwork v1.1.1^83^, ggrepel v0.9.1^84^, VennDiagram v1.6.20^85^ and ComplexHeatmap v2.4.3^86^.

### Gene Set Enrichment Analysis

For each of the pairwise comparisons in each of the clusters, we applied Gene Set Enrichment Analysis (GSEA)^87^ to the complete list of genes in our dataset ordered by the log2FC obtained in the differential expression analysis, using the gseGO function of the package clusterProfiler v3.16.1^88^ with default parameters and the packages AnnotationDbi v1.50.3 and org.Mm.eg.db v3.11.4^89^ for interoperability between different gene annotations. Gene Ontology (GO) biological processes were considered enriched if the obtained permutation test’s p-value adjusted with Benjamini-Hochberg (BH) method was lower than 0.05. The resulting tables were further simplified using clusterProfiler’s simplify function with default parameters and pathways were plotted using Revigo^90^ and ggplot2 v3.3.3.

### Differential transcriptional variability analysis

The tool BASiCS v2.4.0^48,49^, in R v4.1.0, was used to find genes showing different levels of transcriptional variability between conditions in each of the HSC clusters. For each cluster and condition, we fitted the regression between over-dispersion and mean expression using the BASiCS_MCMC function with the recommended settings and without adding spike-in genes. The function BASiCS_TestDE was used to determine which genes are differentially variable between conditions in each HSC cluster, considering as significant those genes with a difference in the residual over-dispersion >0.5 and an expected false discovery rate (FDR) of the posterior probability <0.05. We also calculated the Spearman correlation between the difference in the residual over-dispersion of aged over young and aged over CASIN, as well as for aged over young and CASIN over young. Results were plotted using ggplot2 v3.3.5, ggrepel v0.9.1^84^, patchwork v1.1.1^83^, and VennDiagram v1.6.20^85^.

### Cluster connectivity analysis

We computed Partition-based Graph Abstraction (PAGA)^51^ for each of the conditions using python v3.7.9 and scanpy 1.8.0^91^, after previously computing each neighborhood graph with 30 nearest neighbors and 25 PCs. The pseudotime of cells in the trajectories between interesting pairs of clusters was computed using scanpy, again with 25 PCs. For every trajectory, the package tradeSeq v1.6.0^52^, with R v4.1.0, was used to fit a regression between the expression values and the cells ordered by pseudotime for each gene and each condition (using 6 knots) and to look for genes with different patterns of expression between conditions (with absolute log2FC higher than 0.25 and Wald test’s BH adjusted p-value lower than 0.05). GSEA was applied to the complete list of genes ordered by the Wald statistic obtained in the previous test using the gseGO and simplify functions of clusterProfiler v.4.0.5^92^, with default parameters and positive score type, and packages AnnotationDbi v1.54.1^93^ and org.Mm.eg.db v3.13.0^89^ for intercompatibility between different gene annotations. The genes involved in interesting GO biological processes were clustered according to the similarities in their expression patterns using tradeSeq’s clusterExpressionPatterns function with 30 points and a minimum cluster size of 3.

### Reporting summary

Further information on research design is available in the Nature Research Reporting Summary linked to this article.

## Supplementary information

Supplementary Figures 1-8

Reporting Summary

Supplementary Dataset 1

Supplementary Dataset 2

Supplementary Dataset 3

Supplementary Dataset 4

Supplementary Dataset 5

Supplementary Dataset 6

Supplementary Dataset 7

Supplementary Dataset 8

Supplementary Dataset 9

Supplementary Dataset 10

Supplementary Movie 1

Supplementary Movie 2

## Data Availability

The source data underlying Figs. 1–4 and 6 and Supplementary Figs. 1, 4, 5, and 8e are provided as a Source Data file. Data for Fig. 5 and Supplementary Figs. 6, 7, and 8a–d are provided as Supplementary Datasets. All data and codes are deposited under the repository 10.34810/data163. scRNA-seq data are deposited at GEO (accession number GSE197070). Dilutions and catalog numbers of all commercial antibodies are provided in the Source Data file.
