# Supplementary material for: Transplanting rejuvenated blood stem cells extends lifespan of aged immunocompromised mice
Source: NPJ Regen Med. 2022 Dec 29;7:78. doi: 10.1038/s41536-022-00275-y (PMC9800381; doi:10.1038/s41536-022-00275-y)
Supplement: Supplementary file 1 — Supplementary Figures 1-8 [file 41536_2022_275_MOESM1_ESM.pdf]

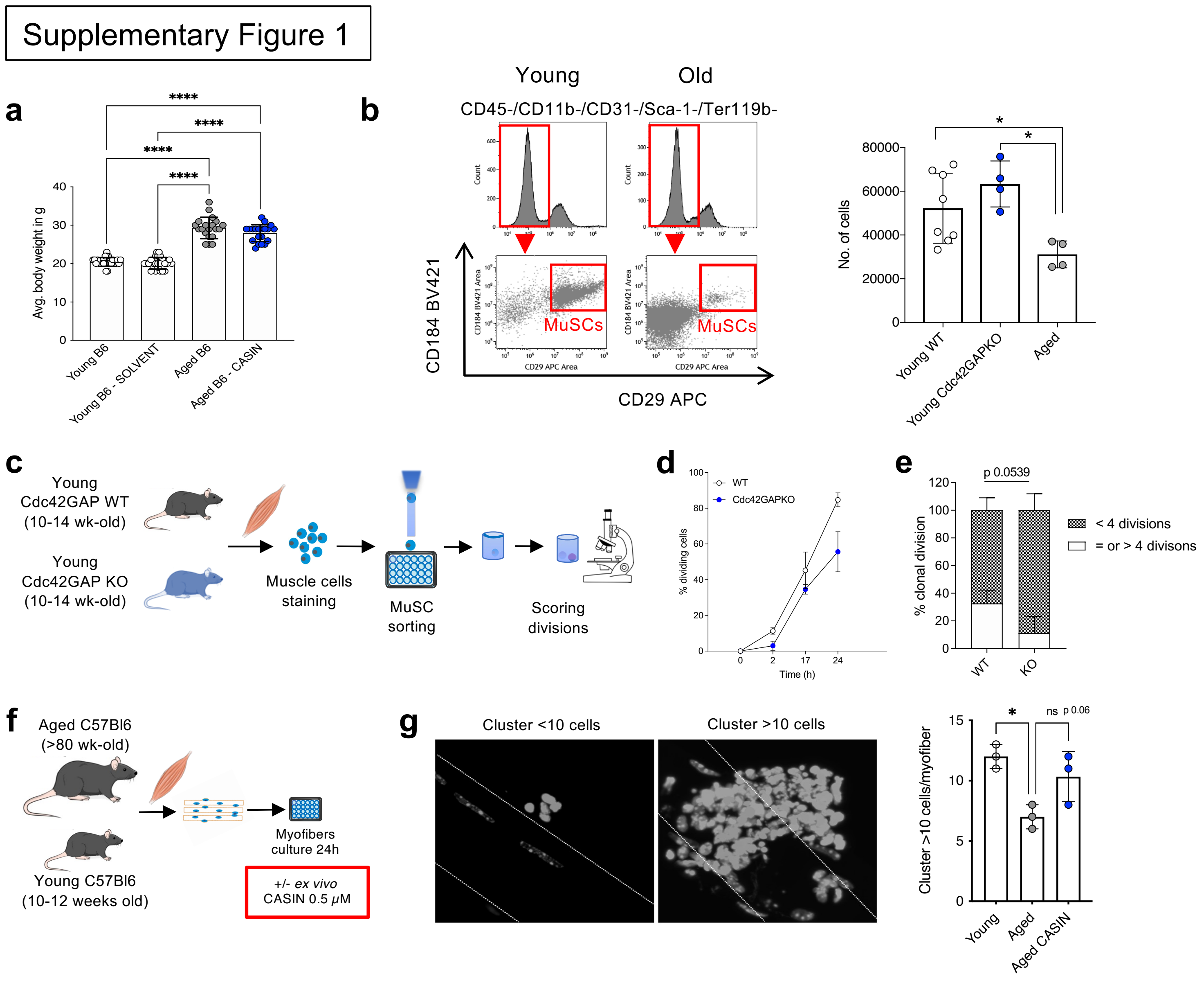

Supplementary Figure 1. MuSC from Cdc42GAP mice show some premature aging-like phenotypes and treating aged MuSCs with CASIN improves stem cell function in vitro. **a** Body weight measurements of young and aged mice before and after injections (Young, n=25; Aged n=20). **b** Representative flow cytometry panels for MuSCs isolated from young and old mice and quantification of MuSCs isolated from muscle tissue (hindlimbs) of young wild-type, Cdc42GAP and aged mice (n=8-4). **c** Experimental scheme of single MuSC isolation for growth kinetics. **d** Kinetic growth curve for MuSC isolated from hindlimbs of young wild-type and Cdc42GAP mice (wild-type, n=2; Cdc42GAP, n=3). **e** Quantification of clonal divisions on day 6 for single MuSCs isolated from young wild-type and Cdc42GAP mice (wild-type, n=3; Cdc42GAP, n=4). **f** Myofiber isolations and in vitro CASIN treatment scheme. **g** Representative images showing the clusters on myofibers after 24h in culture as categorized and relative quantification (n=3 mice). Values are given as mean ±SD. **P* < 0.05, ***P* < 0.01, ****P* < 0.001 and *****P* < 0.0001; two-way ANOVA, multiple comparison Tukey test for **a**, **b** and **g**. Student t test for **e**. Source data are provided as a Source Data file. Mouse cartoon: CreativeBucket:stock.adobe.com. Muscle cartoon: **smart.servier.com**

Supplementary Figure 2

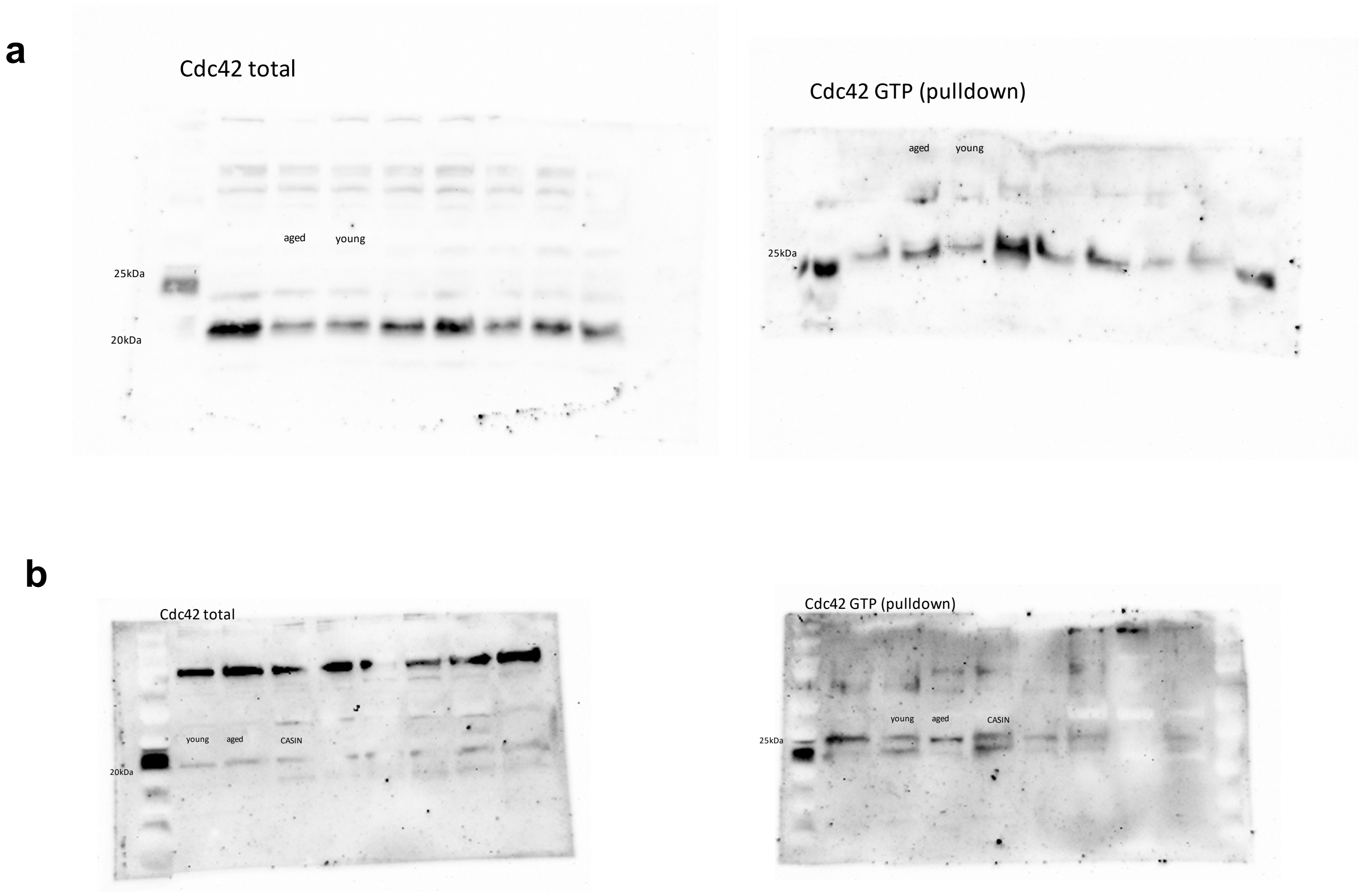

Supplementary Figure 2. Representative western blot and pulldown. a Representative blots for Cdc42 total and Cdc42GTP (pulldown) in cells harvested from the skeletal muscle tissue of young and aged mice. **b** Representative blots for Cdc42 total and Cdc42GTP (pulldown) in cells harvested from the skeletal muscle tissue of young, aged and aged+CASIN mice.

Supplementary Figure 3

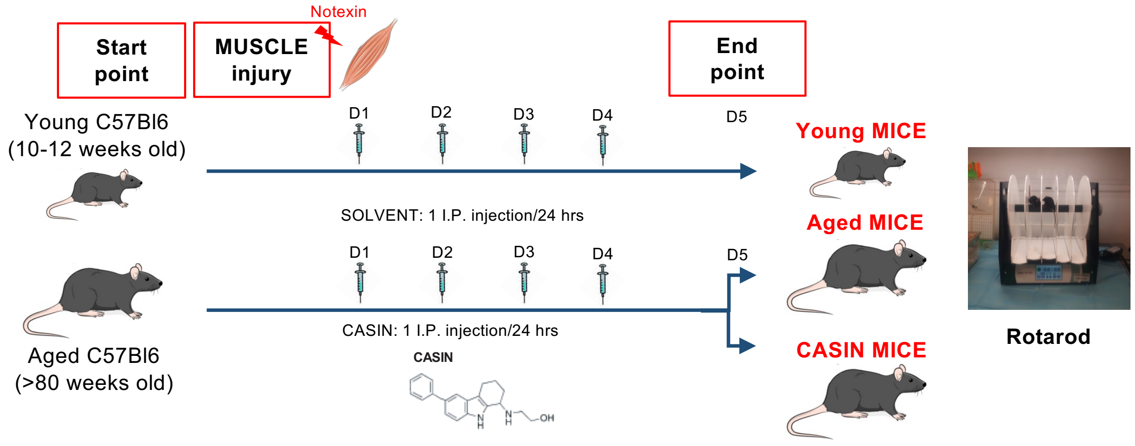

Supplementary Figure 3. Systemic inhibition of Cdc42 activity improves aged mouse fitness and the regenerative potential of aged MuSCs. Cartoon scheme depicting the experimental set up for the rotarod test coupled to the Ntx injury. Young and aged mice were first assayed for their endurance resistance at day 0. Afterwards, mice were injured with Ntx on both legs and half of the aged mice were randomly selected for CASIN treatment for the 4 days following the Ntx injury. On day 5 all mice were assayed again for their endurance resistance on the rotarod. Mouse cartoon: CreativeBucket:stock.adobe.com. Muscle cartoon: **smart.servier.com**

Supplementary Figure 4

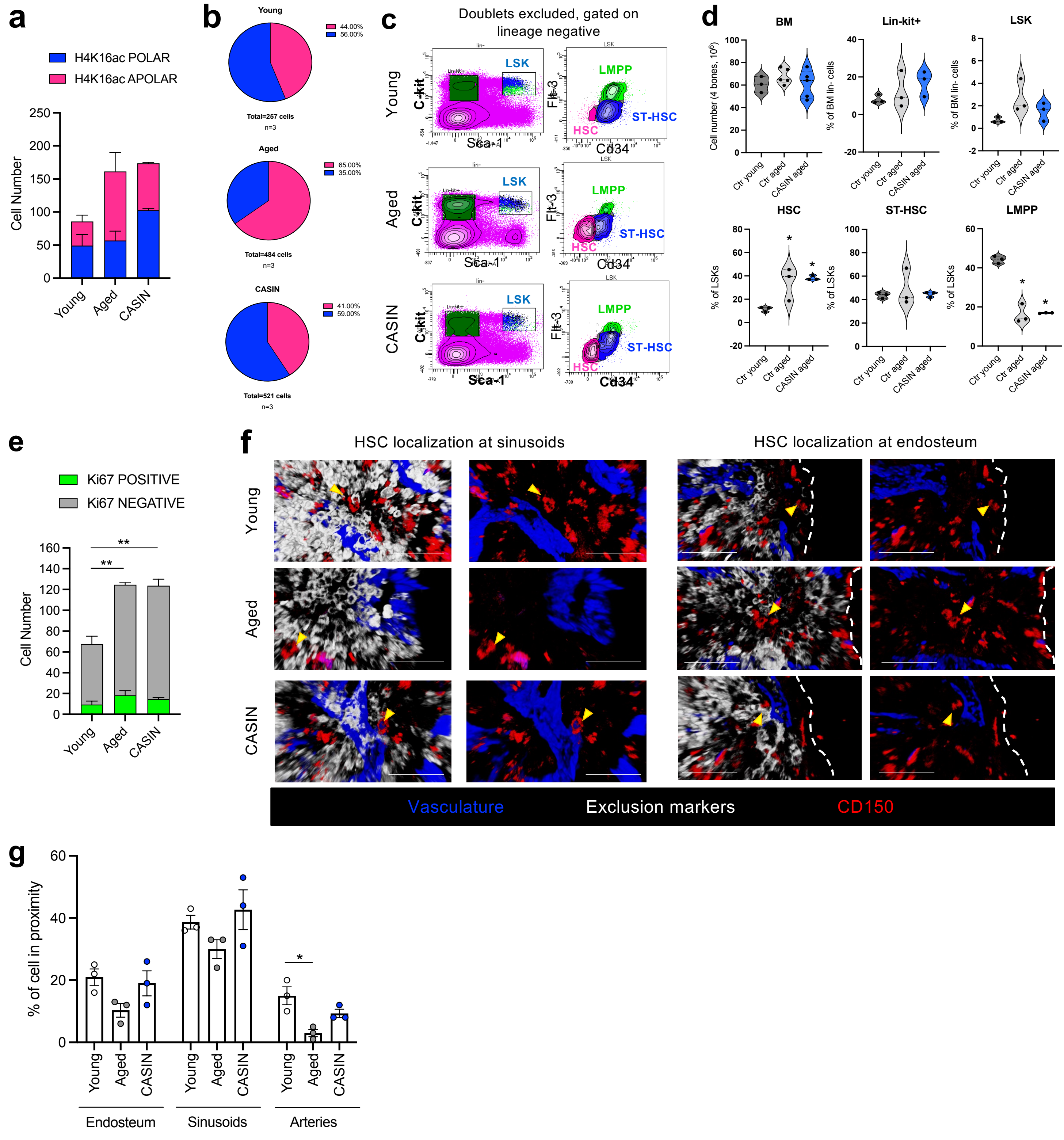

Supplementary Figure 4. Systemic inhibition of Cdc42 activity increases hematopoietic stem cell (HSC) polarity and restores stem cell localization in BM of aged mice. a Quantification of H4K16ac polar and apolar HSC cell number in young, aged and CASIN mice (n=3). **b** Pie chart representation of the percentage of H4K16ac polar and apolar HSCs in young, aged and CASIN mice (n=3). **c** Representative flow cytometry panels and gating strategy for LSKs, LMPPs, ST-HSCs and HSCs from bone marrow cell samples obtained from young, aged and CASIN mice. **d** Number of total BM cells and percentage of Lin-kit+, LSKs, LMPPs, ST-HSCs and HSCs from bone marrow cell samples obtained from young, aged and CASIN mice (n=3). **e** Quantification of Ki67 positive and negative HSC cell number in young, aged and CASIN mice (n=3). **f** HSC proximity to sinusoids and endosteum in young, aged and CASIN mice stained for CD150 (red), vasculature (CD31 and CD144) (blue) and lineage marker and CD48 (white). **g** Percentage of Ki67$^-$ HSCs in proximity to endosteum, sinusoids and arteries. Proximity was evaluated as previously described (see Methods) (n=3). Values are mean ±SE. **P* < 0.05, ***P* < 0.01, ****P* < 0.001; one-way ANOVA, multiple comparison with Tukey test. Source data are provided as a Source Data file.

Supplementary Figure 5

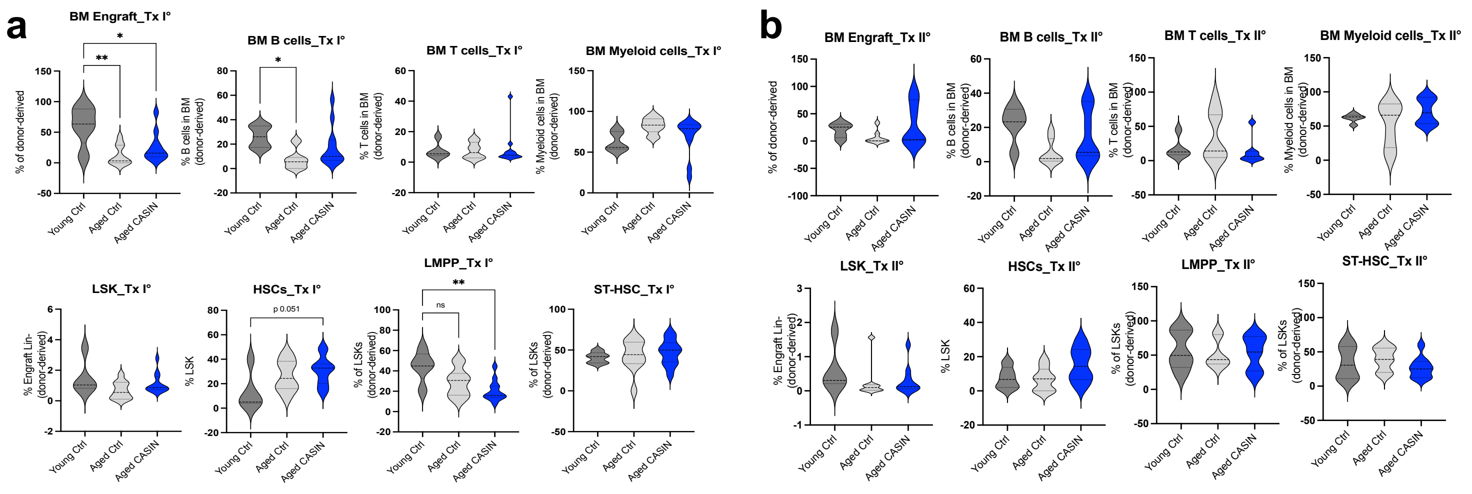

Supplementary Figure 5. Systemic inhibition of Cdc42 activity increases the regenerative potential of aged blood stem cells. a, **b** Flow cytometry quantification of BM profile of recipient mice in primary (Tx I°) and secondary (Tx II°) transplants. (Young, n=5/6; Aged, n=9/12; CASIN, n=7/12). *$P < 0.05$, **$P < 0.01$; one-way ANOVA, multiple comparisons with Dunnett's test. Source data are provided as a Source Data file.

Supplementary Figure 6

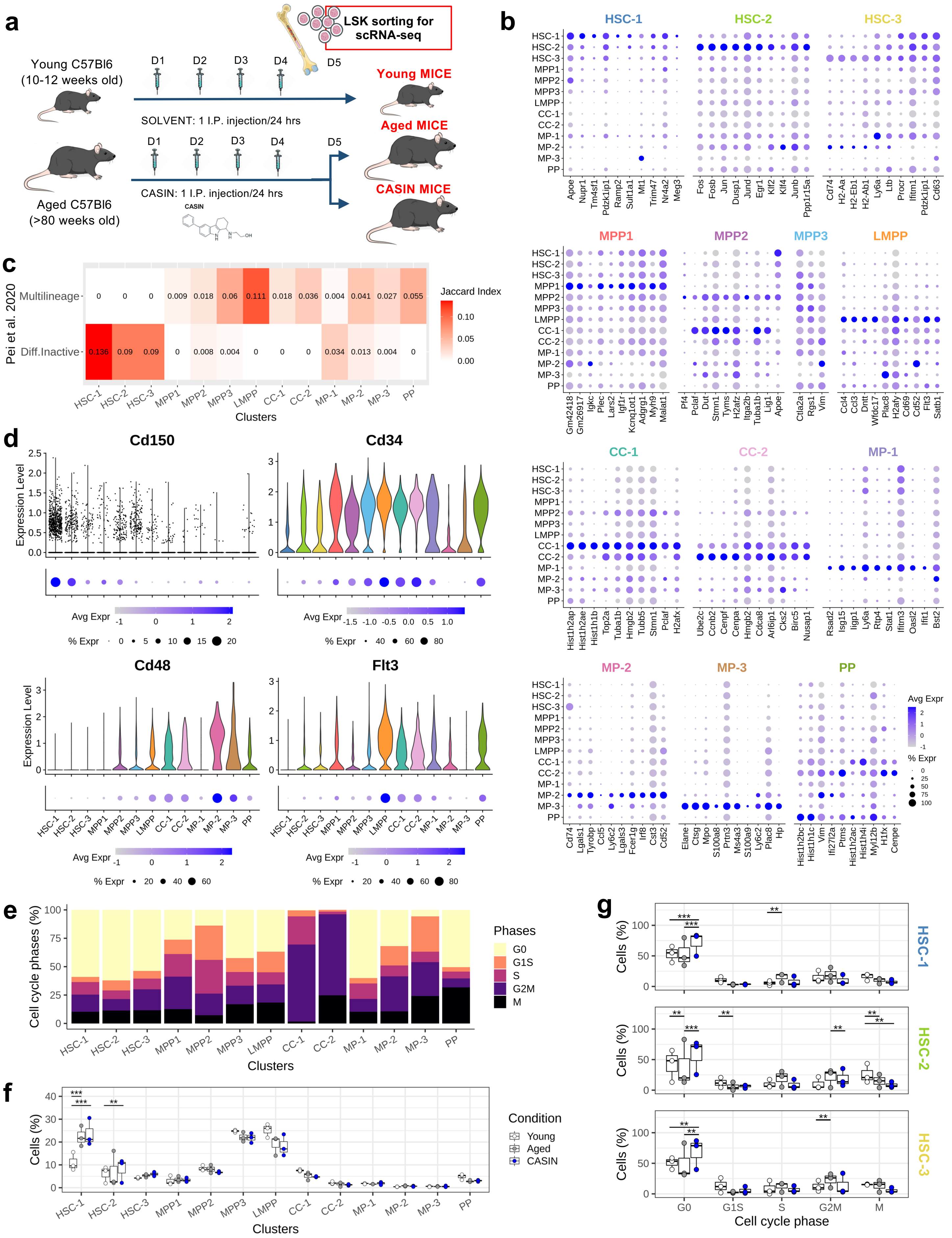

Supplementary Figure 6. scRNAseq profiling of HSCs from young, aged and CASIN mice. a Experimental design.**b** Dot plot representing the expression of the top 10 marker genes with highest log2FC for every cluster. **c** Jaccard indexes for the similarity between the gene signatures defined by Pei et al. for differentiation-inactive and multilineage HSCs and the top 200 marker genes (by log2FC) for every cluster. **d** Violin plots and dot plots for the expression levels of the genes Cd150, Cd34, Cd48 and Flt3 in each cluster. **e** Proportion of cells assigned to each cell cycle phase in every cluster. **f** Proportion of the clusters in every sample, separated by condition. Boxes show median and 25th and 75th percentiles. Statistical credibility (scCODA's effects parameter, EP): *|EP| > 0, **|EP| > 0.25, ***|EP| > 0.5. **g** Proportion of the cell cycle phases in every sample, separated by condition, for each of the HSC clusters. Boxes, statistical credibility and legend as in **f**. Data are provided in Supplementary Datasets 2-3. Mouse cartoon: CreativeBucket:stock.adobe.com. Bone and cells cartoon: **smart.servier.com**

Supplementary Figure 7

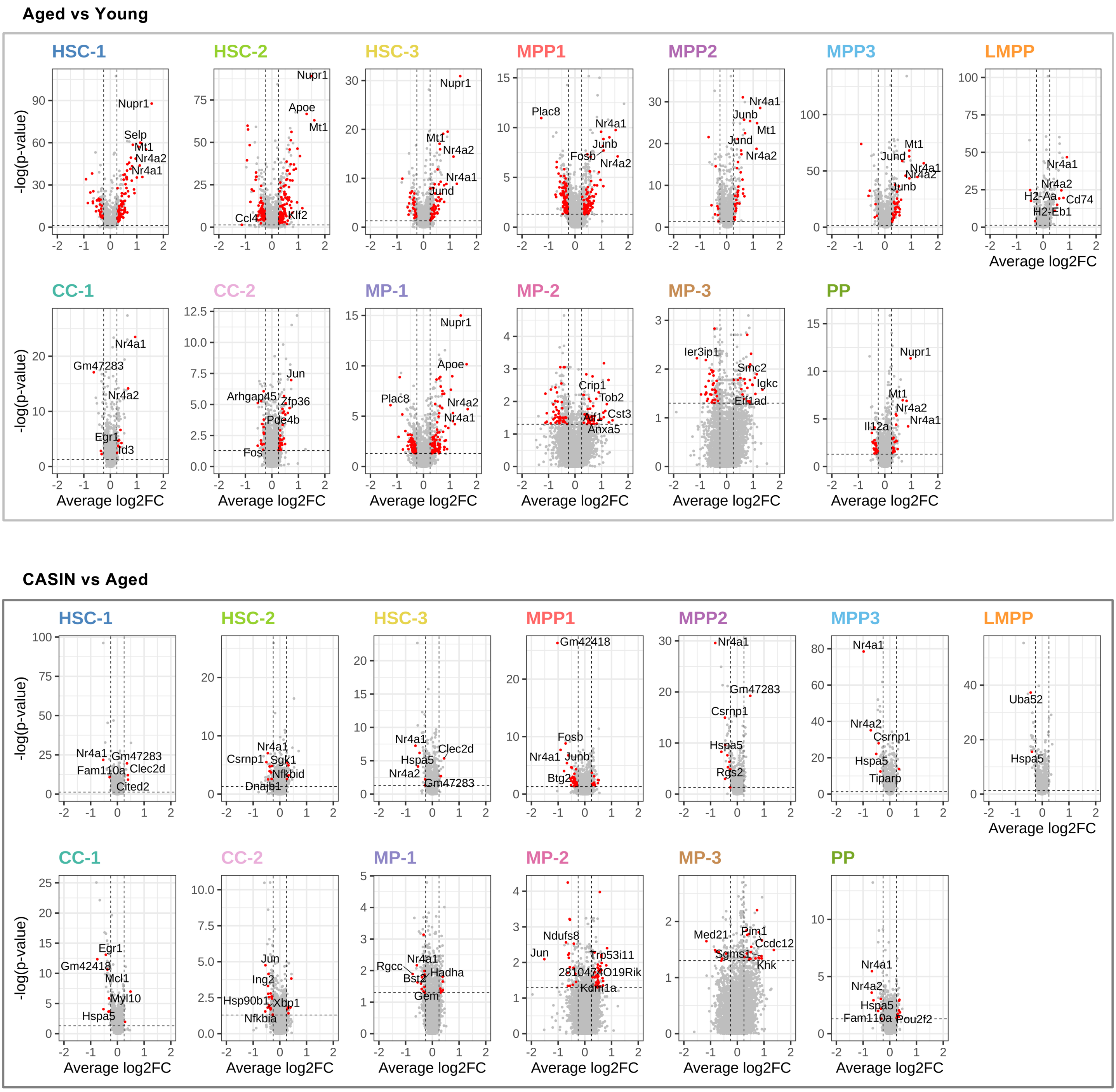

Supplementary Figure 7. Differential gene expression analysis across clusters. Volcano plots of the DE genes in every cluster between aged and young samples and between CASIN and aged samples. Vertical dashed lines: average log2FCs of -0.25 and 0.25. Horizontal dashed line: Tippett's combined p-value of 0.05 for Wilcoxon Rank Sum test. Genes in red are significantly DE in the two sequencing batches. The 5 genes with a higher absolute log2FC are shown. Data are provided in Supplementary Dataset 4.

Supplementary Figure 8

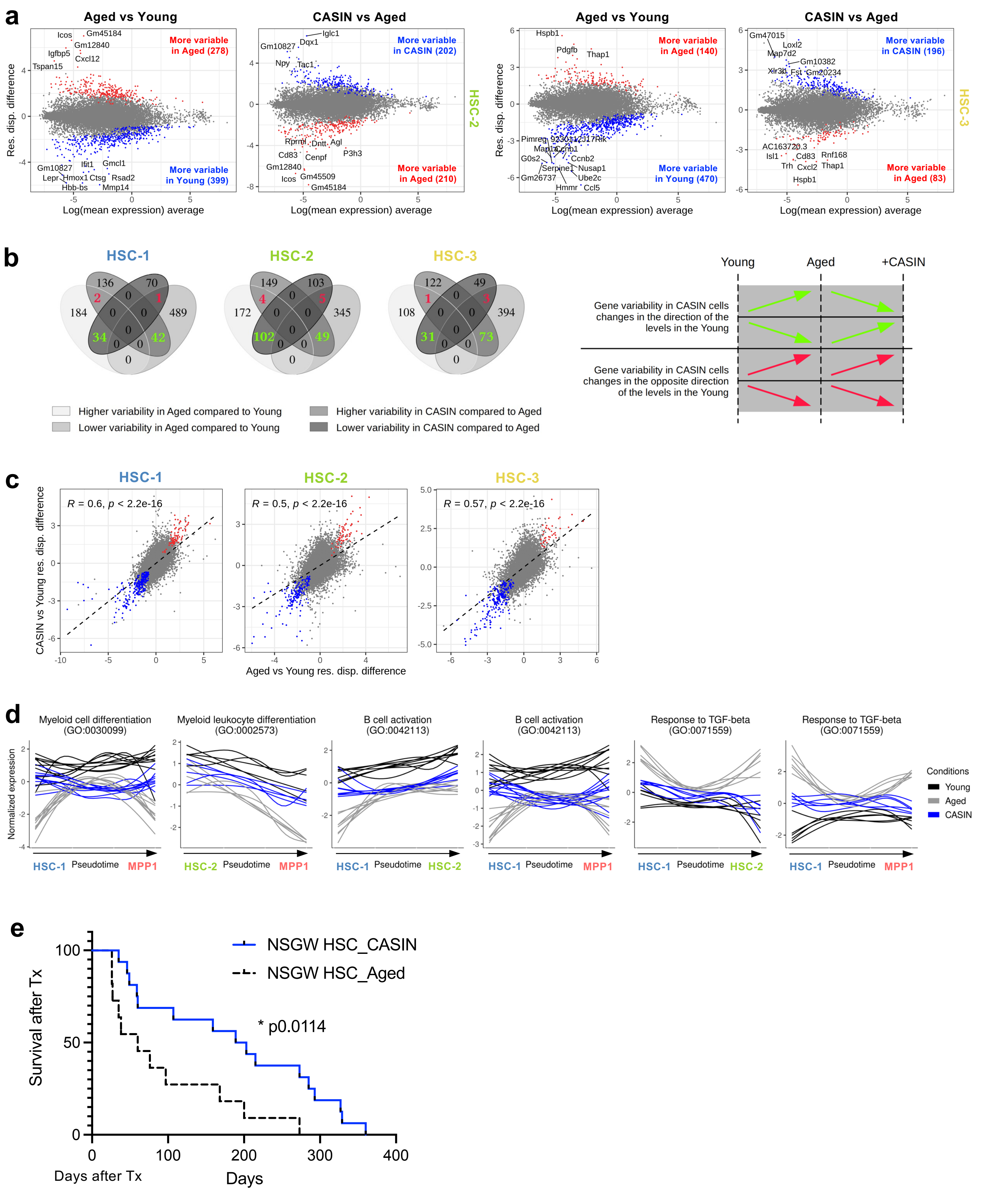

Supplementary Figure 8. CASIN treatment restores a youthful transcriptional heterogeneity in aged HSCs and changes the pattern of expression along pseudotime of several immune related genes. a Residual dispersion difference against average logarithmic mean expression for every gene for the HSC-2 and HSC-3 clusters. Top 15 significantly differentially variable genes shown. **b** Venn diagrams representing the common genes with higher or lower variability in aged compared to young and higher or lower variability in CASIN compared to aged for each HSC cluster. In green, the number of genes whose variability in the CASIN group changes in the direction of the levels in the young mice. In red, the number of genes whose variability in the CASIN group changes in the opposite direction of the levels in the young mice (depicted in the right panel). **c** Spearman correlation of the residual dispersion difference of aged over young and CASIN over young. The genes that are more (red) or less (blue) variable in both comparisons are highlighted. **d** Expression pattern over pseudotime of several genes (Supplementary Dataset 10) involved in the indicated GO processes. **e** Survival after Tx of aged NBGSW mice. (Aged, n=10; CASIN, n=16; Not transplanted, n=19). Survival analysis with Mantel Cox test. Data are provided in Supplementary Datasets 6-10 and as a Source Data file.
