# Supplementary material for: Transplanting rejuvenated blood stem cells extends lifespan of aged immunocompromised mice
Source: NPJ Regen Med. 2022 Dec 29;7:78. doi: 10.1038/s41536-022-00275-y (PMC9800381; doi:10.1038/s41536-022-00275-y)
Supplement: Supplementary file 2 — Reporting Summary [file 41536_2022_275_MOESM2_ESM.pdf]

nature portfolio

Corresponding author(s): Maria Carolina Florian

Last updated by author(s): Dec 2, 2022

Reporting Summary

Nature Portfolio wishes to improve the reproducibility of the work that we publish. This form provides structure for consistency and transparency in reporting. For further information on Nature Portfolio policies, see our Editorial Policies and the Editorial Policy Checklist.

Statistics

For all statistical analyses, confirm that the following items are present in the figure legend, table legend, main text, or Methods section.

n/a	Confirmed	
☐	☒	The exact sample size (*n*) for each experimental group/condition, given as a discrete number and unit of measurement
☐	☒	A statement on whether measurements were taken from distinct samples or whether the same sample was measured repeatedly
☐	☒	The statistical test(s) used AND whether they are one- or two-sided<br>*Only common tests should be described solely by name; describe more complex techniques in the Methods section.*
☐	☒	A description of all covariates tested
☐	☒	A description of any assumptions or corrections, such as tests of normality and adjustment for multiple comparisons
☐	☒	A full description of the statistical parameters including central tendency (e.g. means) or other basic estimates (e.g. regression coefficient) AND variation (e.g. standard deviation) or associated estimates of uncertainty (e.g. confidence intervals)
☒	☐	For null hypothesis testing, the test statistic (e.g. *F*, *t*, *r*) with confidence intervals, effect sizes, degrees of freedom and *P* value noted<br>*Give P values as exact values whenever suitable.*
☒	☐	For Bayesian analysis, information on the choice of priors and Markov chain Monte Carlo settings
☐	☒	For hierarchical and complex designs, identification of the appropriate level for tests and full reporting of outcomes
☒	☐	Estimates of effect sizes (e.g. Cohen's *d*, Pearson's *r*), indicating how they were calculated

Our web collection on statistics for biologists contains articles on many of the points above.

Software and code

Policy information about availability of computer code

Data collection	BD FACSDiva 8.0.1, Volocity v7.0, Zen v8.1, FlowJo v10, Graph Pad Prism v9.4.0
Data analysis	FASTQ to count matrices: Cell Ranger v3.1.0.<br>Main scRNA-seq analysis: R v4.0.2, Bioconductor v3.11, Seurat v4.0.2, clustree v0.4.3, ggplot2 v3.3.3, patchwork v1.1.1, ggrepel v0.9.1, VennDiagram v1.6.20, ComplexHeatmap v2.4.3.<br>GSEA: clusterProfiler v3.16.1, AnnotationDbi v1.50.3, org.Mm.eg.db v3.11.4, Revigo (http://revigo.irb.hr/).<br>Compositional analysis: python v3.8.10, scCODA v0.1.2.<br>Transcriptional variability analysis: R v4.1.0, Bioconductor v3.13, BASiCS v2.4.0, ggplot2 v3.3.5, ggrepel v0.9.1, patchwork v1.1.1, VennDiagram v1.6.20.<br>Cluster connectivity analysis: python v3.7.9, scanpy v1.8.0, R v4.1.0, tradeSeq v1.6.0, clusterProfiler v4.0.5, AnnotationDbi v1.54.1, org.Mm.eg.db v3.13.0.

For manuscripts utilizing custom algorithms or software that are central to the research but not yet described in published literature, software must be made available to editors and reviewers. We strongly encourage code deposition in a community repository (e.g. GitHub). See the Nature Portfolio guidelines for submitting code & software for further information.

Data

Policy information about availability of data

All manuscripts must include a data availability statement. This statement should provide the following information, where applicable:

- Accession codes, unique identifiers, or web links for publicly available datasets
- A description of any restrictions on data availability
- For clinical datasets or third party data, please ensure that the statement adheres to our policy

All data and code are deposited under the repository https://doi.org/10.34810/data163. scRNA-seq data are deposited at GEO (accession number GSE197070).

Human research participants

Policy information about studies involving human research participants and Sex and Gender in Research.

Reporting on sex and gender	N/A
Population characteristics	N/A
Recruitment	N/A
Ethics oversight	N/A

Note that full information on the approval of the study protocol must also be provided in the manuscript.

Field-specific reporting

Please select the one below that is the best fit for your research. If you are not sure, read the appropriate sections before making your selection.

☒ Life sciences ☐ Behavioural & social sciences ☐ Ecological, evolutionary & environmental sciences

For a reference copy of the document with all sections, see nature.com/documents/nr-reporting-summary-flat.pdf

Life sciences study design

All studies must disclose on these points even when the disclosure is negative.

Sample size	Sample size was determined according to the standard deviation of the measured parameter. We used StatMate and the statistical guide of Prism to help with sample size calculation.
Data exclusions	Data were excluded only in the case of clear technical problems in data analysis due to equipment mis-functioning or to error of the operator. in this eventuality, the error was officially reported on the laboratory notebook and the experimental dataset was excluded from the final analysis.
Replication	All the experiments were repeated in completely different experimental days at least twice, for a minimium total number of biological replicates of 3.
Randomization	Mice were randomized for sex but not for age. Operators were not blinded because aged mice require special care and attention. In the case of CASIN treatment, aged mice were randomly allocated to the control or treatment group.
Blinding	The operator was blind during data collection but not for data analysis.

Reporting for specific materials, systems and methods

We require information from authors about some types of materials, experimental systems and methods used in many studies. Here, indicate whether each material, system or method listed is relevant to your study. If you are not sure if a list item applies to your research, read the appropriate section before selecting a response.

Materials & experimental systems

n/a	Involved in the study	
☐	☒	Antibodies
☒	☐	Eukaryotic cell lines
☒	☐	Palaeontology and archaeology
☐	☒	Animals and other organisms
☒	☐	Clinical data
☒	☐	Dual use research of concern

Methods

n/a	Involved in the study	
☒	☐	ChIP-seq
☐	☒	Flow cytometry
☒	☐	MRI-based neuroimaging

Antibodies

Antibodies used

Antigen Host species Working dilution (method) Catalog number Company/Source

Primary
anti-alpha tubulin, Clone YL1/2 rat 1:1000 (IF) ab 6160 Abcam
anti-MyoD C20 rabbit 1:50 (IF) sc-304 SantaCruz Biotechnology
anti-Pax-7 clone PAX7 mouse 1:20 (IF) Pax7-s Developmental Studies Hybridoma Bank
anti-Laminin rabbit 1:100 (IF) L9393-100UL Sigma Aldrich
anti-Cdc42 total rabbit 1:200 (IF) ab64533 Abcam
anti-Cdc42 total rabbit 1:200 (WB) 07-1466 Merk-Millipore
anti-H4K16ac Rabbit 1:200 (WMH) 07-329 Merk-Millipore
FITC-anti-Ki-67, Clone SolA15 Rat 1:100 (WMH) 11-5698-82 eBioscience
anti-CD41-Biotin Rat 1:2500 (WMH) 13-0411-82 eBioscience
anti-CD48-Biotin Armenina Hamster 1:100 (WMH) 13-0481-82 eBioscience
anti-CD11b-Biotin, Clone M1/70 Rat 1:100 (WMH) 13-0112-82 eBioscience
anti-B220-Biotin, Clone RA3-6B2 Rat 1:100 (WMH) 13-0452-82 eBioscience
anti-CD5-Biotin Clone53-7.3 Rat 1:100 (WMH) 13-0051-82 eBioscience
anti-Gr1-Biotin, Clone RB6-8C5 Rat 1:100 (WMH) 13-5931-82 eBioscience
anti-Ter119-Biotin Rat 1:100 (WMH) MA5-17819 eBioscience
anti-CD8a-Biotin, Clone 53-6.7 Rat 1:100 (WMH) 13-0081-82 eBioscience
anti-CD31-APC, Clone MEC13.3 Rat 0,8mL/g 102510 BioLegend
anti-CD144-AF647 Rat 0,32ml/g 138006 BioLegend

Secondary
AF488 anti-rabbit IgG (H+L) donkey 1:500 (IF) 1:300 (WMH) 711-545-152 Jackson Immuno Research Laboratories
AF594 anti-mouse IgG (H+L) donkey 1:500 (IF) 715-585-151 Jackson Immuno Research Laboratories
Cy3 anti-mouse IgG (H+L) donkey 1.1000 (IF) 715-165-151 Jackson Immuno Research Laboratories
AF488 anti-rat IgG (H+L) donkey 1:500 (IF) 712-545-153 Jackson Immuno Research Laboratories
AF647 anti-rabbit IgG (H+L) donkey 1:200 (IF) 712-605-153 Jackson Immuno Research Laboratories
SA-eFluor450 1:60 (WMH) 48-4317-82 eBioscience

FACS Antibodies

Antigen Host species Working dilution (method) Catalog number Company/Source
"Brilliant Violet 421 - CD184 (CXCR4),
Clone L276F12" Rat 1:50 (FACS) 146511 Biolegend
APC - CD29, Clone - eBioHMb1-1 (HMb1-1) Armenian hamster 1:100 (FACS) 17-0291-80 Invitrogen
CD45-Biotin conjugated, Clone - 30-F11 Rat 1:100 (FACS) 103104 Biolegend
CD11b-Biotin conjugated, Clone M1/70 Rat 1:200 (FACS) 13-0112-85 Invitrogen
CD31-Biotin conjugated, Clone 390 Rat 1:50 (FACS) 102404
Sca-1-Biotin conjugated, Clone E13-161.7 Rat 1:1000 (FACS) 122504 Biolegend
Ter119-Biotin conjugated, Clone TER-119 Rat 1:200 (FACS) 13-5921-85 Invitrogen

SA - APCCY7 1:100 (FACS) 554063 BD Pharmingen
SA - FITC 1:100 (FACS) 11-4317-87 BD Pharmingen

Validation

All primary antibodies were validated in previous publications and/or by the producing company according to the indications in the websites.

Animals and other research organisms

Policy information about studies involving animals; ARRIVE guidelines recommended for reporting animal research, and Sex and Gender in Research

Laboratory animals

Young and aged C57BL/6 mice were obtained from the internal divisional stock (derived from mice ob-tained from both The Jackson Laboratory and Janvier), as well as from NIA/Charles River. The NBSGW mice (JAXStock No.026622) were maintained as homozygotes. Cdc42GAP mice were described previ-ously (Wang et al., 2007). All mice were housed in the animal barrier facility under pathogen-free condi-tions at the University of Ulm and at the Biomedical Research Institute of Bellvitge (IDIBELL). Throughout

	the manuscript, young C57BL/6 mice are between 10 and 16 weeks old and aged C57BL/6 mice are at least 80 weeks old. C57BL/6 mice were randomized for sex.
Wild animals	N/A
Reporting on sex	mice were randomized for sex and equal number of males and females mice were used across samples. sex was not analised as independent variable.
Field-collected samples	N/A
Ethics oversight	All mouse experiments were performed in compliance with the ethical regulations according to: 1) the German Law for Welfare of Laboratory Animals and were approved by the Institutional Review Board of the Ulm University, as well as by the state government of Baden-Württemberg, Regierungspraesidium Tuebingen; 2) the Spanish Law for Animal Protection and Welfare Code and were previously approved in the project AR18008/10399 by IDIBELL's Ethical Committee for Animal Experimentation (CEEA-IDIBELL) as well as by Generalitat of Catalunya.

Note that full information on the approval of the study protocol must also be provided in the manuscript.

Flow Cytometry

Plots

Confirm that:

☒ The axis labels state the marker and fluorochrome used (e.g. CD4-FITC).

☒ The axis scales are clearly visible. Include numbers along axes only for bottom left plot of group (a 'group' is an analysis of identical markers).

☒ All plots are contour plots with outliers or pseudocolor plots.

☒ A numerical value for number of cells or percentage (with statistics) is provided.

Methodology

Sample preparation	Mononuclear BM cells were isolated by low-density centrifugation (Histopaque 1083, Sigma) and stained with a cocktail of biotinylated lineage antibodies. PB was freshly collected. Red blood cell were lysed and remaining white blood cells were stained with a cocktail of antibodies. Muscles from both hindlimbs (TA, EDL, gastrocnemius, plantaris, soleus) and/or Notexin-injured TA muscles from young, aged and CASIN-treated aged mice where indicated were harvested. Following mincing and enzymatic digestion with Skeletal Muscle Dissociation Kit mouse and rat (130-098-305, Miltenyi Biotec) and gentleMACS octo dissociator (130-095-937, Miltenyi Biotec), the digested tissue was centrifuged at 1500 RPM at 10 min. The pellet was resuspended with DMEM (high glucose, py-ruvate, 41966029, Invitrogen) +10%FBS (F2442, Sigma Aldrich) and filtered through MACS Smart-strainers 70µm (130-098-462, Miltenyi Biotec) and 30µm (130-098-458, Miltenyi Biotec). The cell sus-pension was stained with lineage negative antibodies for 20 min at 4°C.
Instrument	BD FACS Aria III (BD Bioscience), LSRII flow cytometer (BD Biosciences)
Software	data were collected with Diva software and analysed with either Diva or FlowJo.
Cell population abundance	N/A
Gating strategy	figures provided in the manuscript.

☒ Tick this box to confirm that a figure exemplifying the gating strategy is provided in the Supplementary Information.
